# Seed traits are pleiotropically regulated by the flowering time gene *PERPETUAL FLOWERING 1* (*PEP1*) in the perennial *Arabis alpina*

**DOI:** 10.1111/mec.15034

**Published:** 2019-03-15

**Authors:** Patrick William Hughes, Wim J. J. Soppe, Maria C. Albani

**Affiliations:** ^1^ Max Planck Institute for Plant Breeding Research Cologne Germany; ^2^ Botanical Institute University of Cologne Cologne Germany; ^3^ Institute of Molecular Physiology and Biotechnology of Plants (IMBIO) University of Bonn Bonn Germany; ^4^ Center of Excellence in Plant Sciences (CEPLAS) Düsseldorf Germany; ^5^ Present address: Rijk Zwaan De Lier The Netherlands

**Keywords:** *Arabis alpina*, ecological genetics, flowering, *FLOWERING LOCUS C*, life history evolution, *PERPETUAL FLOWERING 1*, pleiotropy, seed longevity

## Abstract

The life cycles of plants are characterized by two major life history transitions—germination and the initiation of flowering—the timing of which are important determinants of fitness. Unlike annuals, which make the transition from the vegetative to reproductive phase only once, perennials iterate reproduction in successive years. The floral repressor *PERPETUAL FLOWERING 1* (*PEP1*), an ortholog of *FLOWERING LOCUS C*, in the alpine perennial *Arabis alpina* ensures the continuation of vegetative growth after flowering and thereby restricts the duration of the flowering episode. We performed greenhouse and garden experiments to compare flowering phenology, fecundity and seed traits between *A. alpina* accessions that have a functional *PEP1* allele and flower seasonally and *pep1* mutants and accessions that carry lesions in *PEP1* and flower perpetually. In the garden, perpetual genotypes flower asynchronously and show higher winter mortality than seasonal ones. *PEP1 *also pleiotropically regulates seed dormancy and longevity in a way that is functionally divergent from *FLC*. Seeds from perpetual genotypes have shallow dormancy and reduced longevity regardless of whether they after‐ripened in plants grown in the greenhouse or in the experimental garden. These results suggest that perpetual genotypes have higher mortality during winter but compensate by showing higher seedling establishment. Differences in seed traits between seasonal and perpetual genotypes are also coupled with differences in hormone sensitivity and expression of genes involved in hormonal pathways. Our study highlights the existence of pleiotropic regulation of seed traits by hub developmental regulators such as *PEP1*, suggesting that seed and flowering traits in perennial plants might be optimized in a coordinated fashion.

## Abstract 2

https://doi.org/10.1111/mec.15003

## 1 INTRODUCTION

Plant life cycles are characterized by discrete phase transitions, such as the initiation of reproduction and the germination of seeds. The timing of age‐dependent transitions and their coordination with environmental cues strongly determines fitness (Bradford, 2005; Pianka & Parker, 1975; Takada, 1995). Both the timing of seed germination and flowering have evolved in response to predictable cues, such as seasonal thresholds of day length and temperature (Fenner, 1998; Imaizumi & Kay, 2006; Roff, 1996). The identification of genes regulating phenology is an important ongoing task in plant biology. However, the roles that genetic elements play in regulating phenological traits in annuals, which reproduce only once, may differ in perennials, which reproduce many times.

The regulation of flowering in response to a prolonged period of cold (vernalization) is an important phenological trait that differs among annual and perennial Brassicaceae species. In *Arabidopsis thaliana* L., the MADS‐box transcription factor FLOWERING LOCUS C (FLC) regulates flowering in response to vernalization (Michaels & Amasino, 1999; Sheldon, Rouse, Finnegan, Peacock, & Dennis, 2000). It does so by repressing flowering activators, such as *SUPPRESSOR OF OVEREXPRESSION OF CO1* (*SOC1*) and *FLOWERING LOCUS T* (*FT*) (Deng et al., 2011; Helliwell, Wood, Robertson, Peacock, & Dennis, 2006; Mateos et al., 2017; Searle et al., 2006). Prolonged exposure to cold causes epigenetic changes resulting in the stable and irreversible repression of *FLC* mRNA, which in turn permits flowering to occur after vernalization (Sheldon et al., 2000). Among *A. thaliana* accessions, there is variation in the depth and duration of vernalization required for the stable silencing of *FLC*, and intraspecific variation in *FLC* expression between accessions is associated with adaptive changes in flowering time, including local adaptation (Ågren, Oakley, Lundemo, & Schemske, 2017; Mendez‐Vigo, Pico, Ramiro, Martinez‐Zapater, & Alonso‐Blanco, 2011; Shindo et al., 2005). In *Arabis alpina *L.—a perennial herb in the Brassicaceae—the *FLC* ortholog *PERPETUAL FLOWERING 1 *(*PEP1*) also represses flowering prior to vernalization, but the spatiotemporal variation of this repression facilitates *A. alpina*'s perennial life history. After vernalization, *PEP1* expression is high in axillary branches to suppress flowering and ensure the return to vegetative development (Lazaro, Obeng‐Hinneh, & Albani, 2018; Wang et al., 2009). Different *PEP1* alleles have been observed in *A. alpina* accessions possessing independent mutations that render *PEP1* inactive. These mutations result in lack of a vernalization requirement to flower and perpetual flowering (Albani et al., 2012).

Plants face a general trade‐off between vegetative growth and reproduction (Amir & Cohen, 1990; Bolmgren & Cowan, 2008). Optimal control theory models have been developed to predict the optimal time to initiate flowering, and in the absence of extrinsic factors, an optimal strategy for an annual plant is to allocate all resources to reproduction as soon as current reproductive value is higher than the residual reproductive value (Hirschfield & Tinkle, 1975; Williams, 1966). In perennials, the phases of vegetative growth, flowering and seed production are indistinct, and therefore, maximizing fitness requires understanding the vegetative growth–reproduction trade‐off, as well as how early vegetative growth can increase future reproductive opportunities (Iwasa & Cohen, 1989). The expected fitness of future reproduction is therefore determined by intrinsic factors such as the length of flowering duration, the possibility of vegetative reproduction and seed quality.

Seed germination is a second important developmental transition undertaken by plants. The timing of germination is strongly regulated by seed dormancy mechanisms. Seed dormancy is the ability of viable seeds to abstain from germination despite favourable conditions and is an innate quality that is characterized by unresponsiveness to signals that promote germination (Bewley, 1997). Seed dormancy determines fitness, since seeds with shallow dormancy may germinate too early in the growing season, whereas seeds with high dormancy may germinate too late (Finch‐Savage & Leubner‐Metzger, 2006). Dormancy also allows plants to establish multiyear seed banks and thereby to spread reproductive risk among many seasons (Venable & Brown, 1988). Plant phytohormones such as abscisic acid (ABA) and gibberellic acid (GA) are the primary regulators of seed dormancy. ABA inhibits germination, whereas GA promotes germination, and together, these hormones antagonistically exert control over seed dormancy (Footitt, Douterelo‐Soler, Clay, & Finch‐Savage, 2011).

Seed longevity denotes the ability to retain viability despite ageing‐related stresses such as oxidation and genetic degradation. Longevity is also a critical life history trait, since seed survival is a major factor in the eco‐evolutionary dynamics of seed banks. Predictable decreases in seed viability occur through time, and longevity may also be subject to significant intraspecific variation (Clerkx, Blankestijn‐De Vries, Ruys, Groot, & Koornneef, 2004; Sletvold & Agren, 2015). Many factors affect seed longevity, but plants have two main strategies to avoid the loss of seed viability: protection and repair (Sano et al., 2016). ABA may also play an important role in regulating seed longevity; in *A. thaliana*, *abi1‐5* mutants, which have compromised endogenous ABA biosynthesis, have lower viability after four years of natural ageing (Clerkx, Blankestijn‐De Vries et al., 2004).

Although *FLC* primarily regulates flowering time, *FLC* and *FLC* orthologs have also been shown to pleiotropically regulate other traits, including seed dormancy (Chen et al., 2014; Chiang, Barua, Kramer, Amasino, & Donohue, 2009; Van Tienderen, Hammad, & Zwaal, 1996), inflorescence branching (Huang, Ding, Effgen, Turck, & Koornneef, 2013), circadian rhythm (Edwards et al., 2006), drought resistance (McKay, Richards, & Mitchell‐Olds, 2003) and leaf shape (Willmann & Poethig, 2011). Pleiotropy—the regulation of multiple phenotypes by a single gene—is a ubiquitous feature of the genetic structure of organisms and has both direct and indirect forms. Direct pleiotropy occurs when a single gene is involved in separate molecular processes that give rise to independent phenotypes, while indirect pleiotropy results from a gene affecting multiple phenotypes via molecular pathways that may be diffuse or overlapping (Caspari, 1952; Hodgkin, 1998).

Here, we report that *PEP1* affects plant and seed mortality as well as seed dormancy in a way that is functionally divergent from the role *FLC* plays in regulating seed traits in *A. thaliana*. We demonstrate that *pep1* mutants and perpetual flowering accessions show lower seed dormancy and longevity, reduced sensitivity to exogenous ABA and altered expression of genes conferring ABA sensitivity, including *ABSCISIC ACID INSENSITIVE (ABI) 3* and *5*. To our knowledge, this is the first study demonstrating a pleiotropic link between flowering time genes and seed dormancy or longevity has been found in a perennial species.

## 2 MATERIALS AND METHODS

### 2.1 Plant material

*Arabis alpina* L. (Brassicaceae) is an herbaceous mat‐forming perennial present in alpine and subalpine habitats across Europe, North and East Africa, Western Asia and North America (Assefa, Erich, Taberlet, Nemonissa, & Brochmann, 2007; Ehrich et al., 2007). In Europe, *A. alpina* shows clines of variation in mating system (outcrossing vs. selfing) across much of this range, and seed germination and seedling establishment occur throughout the growing season (Tedder et al. 2015; Torang et al 2015; Laenen et al. 2018). In addition to reproduction through seeds, plants can also propagate clonally by stoloniferous growth (Buehler, Graf, Holderegger, & Gugerli, 2012; Torang et al. 2015). Its perennial life history is characterized by a vegetative phase (during which plants are not able to respond to flowering inductive stimuli) and the ability to keep vegetative growth after flowering. Plants initiate flower buds in response to (and during) prolonged cold and flower very rapidly after snow melt (Lazaro et al., 2018; Torang et al. 2015; Wang et al., 2009). *A. alpina* accessions show two different flowering behaviours (seasonal and perpetual) depending on the duration of the flowering episode and the requirement of prolonged cold to flower. Seasonal flowering accessions require exposure to prolonged cold to flower and restrict the duration of the flowering episode. Perpetual flowering accessions do not require cold to flower and have an extended flowering episode. Natural variation in flowering behaviour and the requirement to flower can be explained by allelic differences in the floral repressor *PEP1* with perpetual flowering accessions carrying lesions in *PEP1* (Albani et al., 2012).

In this study, we used plant material that was characterized by Albani et al. (2012). This material included six natural accessions of *A. alpina *that were sourced from different mountain habitats throughout Europe by the collectors listed in Supporting information Table S1 of Albani et al. (2012). These accessions included three seasonal flowering accessions (Paj, Ara and Sty) and three perpetual flowering accessions (Dor, Tot and Wca). As per Albani et al. (2012), the Ara and Paj accessions were sourced from the Valle de Arán (Catalan: Val D'Aran) in the Spanish Pyrenees and the Cantabrian mountains (at an altitude of 1,400 m), respectively. The Dorfertal (Dor) accession originated from the High Tauern National Park (1,650 m) in the Austrian East Tyrol. The Totes Gebirge (Tot), West Carpathian (Wca) and South Tyrol (Sty) accessions were originally sourced from the Totes Gebirge (Austria), West Carpathian (Austria) and South Tyrol (Italy) mountain ranges (1,600 m), respectively. To better examine the effect of PEP1 on flowering and seed traits, we also included the *pep1‐1 and pep1‐2* mutant alleles. These genotypes were derived from a mutagenesis screen of the accession Paj and have also been described previously (Nordström et al., 2013; Wang et al., 2009).

### 2.2 Plant growth and phenotyping in the greenhouse and garden

Sterilized seeds were stratified (at 4°C for 48 hr), then germinated in filter paper lined Petri plates for four days in a growth chamber providing a 16 hr photoperiod of fluorescent light at 23°C ± 0.1°C. Following germination, seeds were sown in 9 × 9cm pots. Plants were grown in long days (LD; 16 hr:8 hr day:night) in a climate‐controlled greenhouse until ready for vernalization. To avoid precocious flowering of the accessions that do not require vernalization to flower, seasonal accessions were grown for eight weeks, and perpetual genotypes for five weeks prior to vernalization. Subsequently, the plants were split into different groups that will either experience cold in a controlled environment chamber or outside in a garden. Plants for greenhouse replicate experiments 1 and 2 were vernalized in a cold chamber at 4°C under short days (8 hr:16 day:night). The Greenhouse Replicate Experiment 1 cohort was vernalized for 15 weeks and the Greenhouse Replicate Experiment 2 cohort for 12 weeks. For the garden experiment, plants were transferred to the experimental garden on 15–16 October 2015 at the MPIPZ campus in Cologne, Germany. For Greenhouse Experiments 1 and 2, 18–24 plants were used per genotype. For the experimental garden experiment, 16–23 plants were used per accession and assigned to random locations among seven rows of 20–24 plants each.

For Greenhouse Experiments 1 and 2, after vernalization plants were transferred back to a long day greenhouse and were phenotyped once per week for the following traits: plant height, number of flowers on the main stalk, number of branches on the main stalk, number of siliques on main stalk, number of flowers on axillary shoots and number of siliques on axillary shoots. The main stalk of each plant was identified as the first, largest flowering stalk of the plant which senesced much earlier than other inflorescences and had a larger stem width. Plants in the experimental garden were phenotyped in the same way, with additional survivorship determinations recorded after the end (15 March) of each winter. Survivorship estimates report a proportion of plants surviving both winters over the two‐year experiment in the experimental garden (i.e., a single replicate). We used an ONSET HOBO U23 datalogger (Onset Computer Corporation: http://www.onsetcomp.com) to record daily min/max temperature at ground level in the garden, as well as ambient humidity. Day length was calculated using the NOAA Earth System Research Laboratory Solar Calculator (https://www.esrl.noaa.gov/gmd/grad/solcalc/) using the latitude and longitude described above. Temperature data were analysed using the WeatherData package in r.

For both greenhouse and garden plants, seeds within the same cohort were harvested at the same time by selecting siliques that were formed within a five‐day window. For Greenhouse Experiment 1, we harvested siliques at 73 days after the end of vernalization and for Greenhouse Experiment 1 at 75 days after the end of vernalization. Plants flowered less rapidly in the garden, and therefore, seed sampling was performed 125 days after the last temperature drop below 0°C (i.e., 125 days after 15 March, so seeds were collected on 18–20 July). All seeds from marked siliques were harvested within five days of silique maturation. Because after‐ripening is associated with the loss of sensitivity to gibberellic acid (GA), dormancy germination trials and the GA hormone sensitivity trial were begun within four days of seed harvest. Controlled deterioration tests and the paclobutrazol (PBZ) and abscisic acid (ABA) sensitivity trials were begun after after‐ripening had permitted dormancy release.

### 2.3 Flowering schedule in the garden

Critical dates were recorded to characterize the flowering schedules of plants grown in the experimental garden. Plants were regularly phenotyped to determine the date of the onset of flowering (i.e., the number of days from 1 January 2016 to the emergence of the first flower of 2016), the date of the end of flowering (i.e., the number of days from 1 January 2016 to the emergence of the last flower of 2016), the duration of flowering (calculated as the number of days between the onset and end of flowering) and the date of mean flowering (determined as the date by which the 50th percentile flower was produced; for a plant with 10 total flowers, this would be the day when the 5th flower was produced). Mean dates and durations were determined for all accessions and were statistically analysed using a nested mixed model as described below.

### 2.4 Assessments of seed traits

Seed dormancy was assessed by conducting germination assays at different time points as seeds after‐ripened. We made 14 assessments, once per week from 0 to 13 weeks after seed harvest. All assays were performed using four to six independent biological replicates for each accession; six independent biological replicates were used for Paj and the *pep1‐1* mutant, and four for all other accessions. Each of these biological replicates was assayed by four technical replicates for each time point tested. For each assay, a sample of 30–80 sterilized seeds was placed on moistened filter paper in 5cm Petri plates and germinated without stratification in a climate chamber programmed for a 25°C:20°C day:night temperature regime with a 12 hr:12 hr day:night photoperiod cycle. Photographs of the plate were taken seven days after the start of germination, and seed counts were performed in triplicate from these photos. The number of days of dry storage (i.e., after‐ripening) required for 50% germination (DSDS50) was computed by modelling time to germination using a GLM with a logit link (see: Hurtado et al., 2012), and the DSDS50 was the interpolated point at which the germination rate of the seed sample was 50% (He et al., 2014; Hurtado et al., 2012; Joosen et al., 2010).

To assess endogenous differences in seed longevity under controlled conditions, we subjected seeds to artificial ageing using a controlled deterioration test (CDT). To artificially stress seeds, open PCR tubes containing sterilized seeds were placed in an airtight box containing a saturated salt (KCl) solution that fixed the ambient humidity at 83%. Three replicate seed samples were then incubated at 37°C for 0, 1, 2, 3, 4, 6, 8, 10, 12 and 14 days. After incubation, seeds were stratified for 48 hr at 4°C then germinated in a climate chamber programmed for a 25°C:20°C day:night temperature regimen with 12 hr:12 hr day:night photoperiod cycle. Photographs of seeds were produced 10d after germination, and seed counts were performed in triplicate from these photographs. We computed the half‐viability period (P50) for all accessions tested, using a method previously described (Nagel et al., 2016). All assays were performed using four to six independent biological replicates for each accession; six independent biological replicates were used for Paj and the *pep1‐1* mutant, and four for all other accessions. Each biological replicate was assayed by four technical replicates at each time point.

To assess differences in seed sensitivity to exogenous plant hormones, we conducted a series of seed hormone sensitivity trials for assessing seeds from all genotypes and experimental conditions. We conducted a hormone sensitivity trial on seeds collected just after harvest for gibberellic acid (GA4+7), and trials for seeds collected 15 weeks after harvest for abscisic acid (ABA) and paclobutrazol (PBZ). For each trial, samples of 30–80 sterilized seeds from four biological replicates per accession were placed on dry filter paper in 5 cm Petri plates and 1.5 ml of purified water (produced by a Millipore Milli‐Q water purification system) containing a given concentration of GA, ABA or PBZ. Seeds were then germinated without stratification in a climate chamber programmed for a 25°C:20°C day:night temperature regime with a 12 hr:12 hr photoperiod cycle for 10 days. All assays were performed using four to six independent biological replicates for each accession; six independent biological replicates were used for Paj and the *pep1‐1* mutant, and four for *pep1‐2*. Each biological replicate was assayed by four technical replicates at each concentration level for all hormonal treatments.

### 2.5 Analyses of gene expression

Relative expression of target genes was determined using RT‐qPCR. Total RNA was extracted from dry and 24‐hr imbibed seeds using a QIAGEN RNeasy RNA purification kit, and cDNA synthesis was carried out using a QIAGEN Quantitect Reverse Transcription kit. qPCR trials were performed in 96‐well plates on a Bio‐Rad CFX96 Touch Real‐Time PCR system. For each gene of interest, we performed PCR using 150 ng of cDNA and 0.5 μM F + R qPCR primers in 10 μl reaction volumes of master mix from a Bio‐Rad IQ SYBR Green Supermix kit. Observed Ct values were standardized using two housekeeping genes known to be stably expressed in seeds, *AaKU70* (an ortholog of At1g16970 in *A. alpina*) and *AaWU40* (an ortholog of At2g43770; Dekkers et al., 2012). These genes were selected for their stability and efficiency based on a preliminary assessment of a range of housekeeping genes. Relative expression was determined using the comparative C_T_ method. Mean values were determined using three technical replicates each of four biological replicates, and asymmetrical relative error was computed using the method of Livak and Schmittgen (2001).

We tested the expression levels of a variety of genes that may affect seed traits. Specifically, we tested the expression of *PEP1*, the *A. alpina* ortholog of the seed dormancy regulator *DELAY OF GERMINATION 1* (*AaDOG1*) and the *A. alpina* orthologs of genes involved in hormone biosynthesis or signalling that have been shown to play a role in seed dormancy in *A. thaliana*. These included genes linked to ABA such as the 9‐cis‐epoxycarotenoid dioxygenases (*AaNCED6* and *AaNCED9*)*, *the cytochrome P450 CYP707A genes *(AaCYP707A1* and *AaCYP707A2*) and the ABA‐*INSENSITIVE* genes (*AaABI2*, *AaABI3*, *AaABI4* and *AaABI5*) but also genes associated with GA such as the gibberellin 2‐oxidases (*AaGA2ox1*, *AaGA2ox2*, *AaGA2ox3*, *AaGA2ox4*, *AaGA2ox6 *and *AaGA2ox8*), the gibberellin 3‐oxidases (*AaGA3ox1 and AaGA3ox2*), the gibberellin 20‐oxidases (*AaGA20ox2* and *AaGA20ox4*), the protein phosphatase *SHEWANELLA‐LIKE PROTEIN 2 *(*AaSLP2*) and DELLA *RGA‐like 2* (*AaRGL2*). A complete list of the primers used for qPCR is shown in Supporting information Table S1, and the genes referred to in this article can be found in the GenBank/EMBL databases under the accession numbers shown in Supporting information Table S2.

### 2.6 Statistical analyses

To test whether *PEP1* was associated with significant differences in the allocation of reproductive effort, we used ANOVAs to test whether accession groups—that is seasonal accessions (Ara, Paj and Sty), perpetual accessions (Dor, Tot and Wca) and *pep1* mutants (*pep1‐1* and *pep1‐2*)—differed in the proportion of total reproductive effort (i.e., the total number of siliques produced) allocated to the main stalk. Models analysing data from the greenhouse population (which included data from both Replicate Experiments 1 and 2) included one response variable (i.e., the proportion of reproductive effort allocated to the main stalk) and three fixed effects (accession group, replicate—i.e., Replicate Experiment 1 or 2—and their interaction), while models analysing data from the garden population included only accession group as a fixed effect. Where accession group was a significant predictor of the response, we used Tukey HSD tests to conduct pairwise comparisons between accession groups; these HSD tests used false discovery rate (FDR) corrections to ensure that the global alpha remained 0.05. Given the particular usefulness of examining the phenotypic differences between the Paj accession and the *pep1‐1* and *pep1‐2* mutants, we conducted similar pairwise comparisons between these particular genotypes for all response variables. We ensured that residual variation was homoscedastic and that all models fit the data by examining residual plots.

The same factorial/one‐way ANOVA approach combined with post hoc FDR‐corrected pairwise comparisons between means of accession groups and genotypes (i.e., Paj/*pep1‐1*/*pep1‐2*) was used to determine whether accession group was a significant predictor of other flowering and seed traits we measured in both the greenhouse and garden populations, as well as whether significant differences existed between accession group and genotype means. For instance, this approach was used to determine whether accession group was a significant predictor of flowering traits in the experimental garden population, including (a) the mean date of the onset of flowering (measured in days elapsed since 1 January 2016); (b) the mean date of the end of flowering; (c) the duration of flowering; and/or (d) the date of mean flowering (measured as the date on which the 50th percentile flower was produced). We also used this approach to assess differences in survivorship among accession groups in the experimental garden. To analyse seed trait data from the greenhouse and the experimental garden, we used the factorial and one‐way ANOVA approaches, respectively, to predict whether accession group was a significant predictor of seed dormancy (i.e., DSDS50) and longevity (i.e., P50). Post hoc tests were used to evaluate the significant differences between accession group and genotype means for these traits. We used slightly different models to analyse germination tests after natural ageing or at different temperature treatments. To determine whether genotype was a significant predictor of differences in germination after natural ageing, we used an ANCOVA model that included genotype (Paj or *pep1‐1*) as a fixed effect and the duration of natural ageing as a covariate (as well as their interaction), to predict differences in seed germination rate. To determine whether genotype was a significant predictor of seed germination rate at different germination temperatures, we used a factorial ANOVA model that included genotype, germination temperature and their interaction as fixed effects. Finally, we used one‐way ANOVAs to test whether genotype (i.e., Paj, *pep1‐1* or *pep1‐2*) was a significant predictor of seed sensitivity to plant hormones, including ABA, GA and paclobutrazol. Separate seed trait tests were conducted on greenhouse and experimental garden data. All statistical analyses were carried out in r version 3.5.0.

## 3 RESULTS

### 3.1 *PEP1* influences flowering traits and plant mortality

To assess the differences in reproductive output between plants with and without functional *PEP1* alleles, we compared flowering traits among seasonal and perpetual accessions and mutants. Accessions were analysed in three groups (“accession groups”): a seasonal accession group (containing plants from the accessions Paj, Ara and Sty, which have a functional *PEP1*), a perpetual accession group (containing plants from the accessions Dor, Tot and Wca, which carry lesions in *PEP1*) and a mutant group (containing the *pep1‐1* and *pep1‐2* mutants, two mutant alleles of *PEP1*). To characterize flowering behaviour, we examined flowering in different accession groups, as well as the total number of flowers produced and the proportion of flowers present on the main stalk compared to side branches.

**Figure 1:**
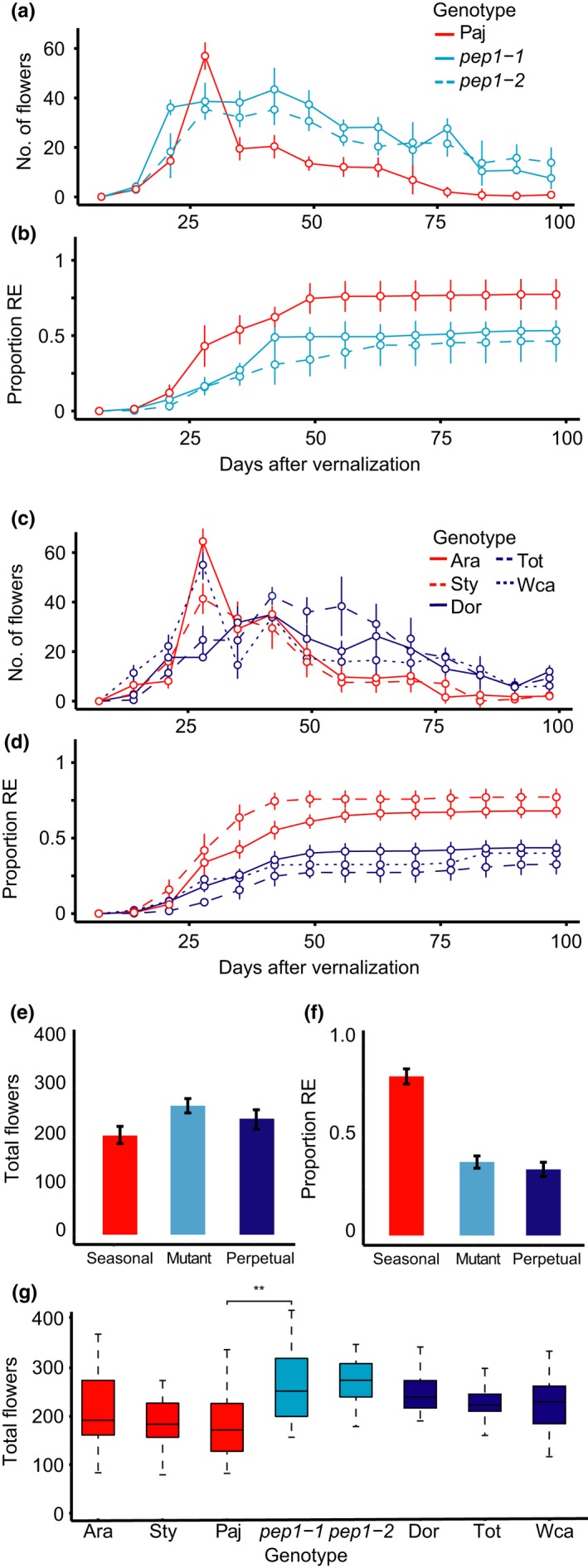
Perpetual flowering accessions and *pep1* mutants show asynchronous flowering and produce more siliques compared to seasonal flowering accessions in a greenhouse. Phenotypes measured in plants after vernalization. (a) Flower production in Pajares (Paj), *pep1‐1* and *pep1‐2; *(b) proportion of total reproductive effort (number of siliques) produced on the main inflorescence in Paj, *pep1‐1* and *pep1‐2*; (c) flower production in seasonal and perpetual accessions; (d) proportion of total reproductive effort produced on the main inflorescence in seasonal and perpetual accessions; (e) a bar chart showing the mean number of flowers produced by accession group; (f) a bar chart showing the mean proportion of reproductive effort produced on the main inflorescence by accession group. For a–f, error bars indicate *SEM*. Also shown: (g) a box‐and‐whisker plot showing the mean total number of siliques produced by all accessions and mutants. Boxes indicate the second and third quartiles of the data, and bars indicate the range. Seasonal accessions (Paj, Ara and Sty) and the seasonal accession group are shown in red, perpetual accessions (Dor, Tot and Wca) and the perpetual accession group in blue, and mutants (*pep1‐1* and *pep1‐2*) and the mutant genotype group in cyan. *N* = 18–24 per accession. Comparisons report Tukey HSD tests at **p < 0.01 (corrected for FDR) [Colour figure can be viewed at http://wileyonlinelibrary.com]

In the greenhouse, both Paj plants and *pep1‐1* and *pep1‐2* plants began to flower approximately a week after the end of vernalization (EV; Figure 1a). Paj plants maximized the rate of flowering at 28 days after EV, then tapered off, ceasing flowering by 77–84 days after EV. Paj plants also had a relatively high proportion of their reproductive effort allocated to the main stalk (mean = 0.75, *SE* = 0.03; Figure 1b; Supporting information Table S3). In contrast, *pep1‐1* and *pep1‐2* plants produced flowers continuously until the end of the experiment (98 days after EV) and showed a lower proportion of their reproductive effort allocated to the main stalk (*pep1‐1* mean = 0.29, *SE* = 0.02; *pep1‐2* mean = 0.34, *SE* = 0.02; Supporting information Table S3). Similarly, other accessions in the seasonal accession group (Ara and Sty) showed an early peak in flowering and a higher proportion of reproductive effort on the main inflorescence, while accessions in the perpetual accession group (Dor, Tot and Wca) continued flowering throughout the experiment and showed a lower proportion of reproductive effort on the main inflorescence (Figure 1c, d).

Statistical analysis revealed that accession group was a strong predictor of the total number of flowers produced (Table 1), with seasonal accessions (mean = 197.6, *SE* = 7.3) producing significantly fewer flowers than perpetual accessions (mean = 220.3, *SE* = 5.9) or mutants (mean = 257.5, *SE* = 5.6; Figure 1e; Supporting information Table S4). However, mutant genotypes and perpetual accessions also significantly differed in the mean number of flowers produced (Supporting information Table S4). A separate analysis showed that there was no difference in the mean number of seeds produced per silique in Paj (mean = 22.6 seeds, *SE* = 3.94) and *pep1‐1 *(mean = 22.9, *SE* = 4.52) plants (Supporting information Figure S1). Next, we examined the proportion of reproductive effort invested in the inflorescence on the main shoot axis compared to axillary branches. An ANOVA revealed that seasonal accessions (mean = 0.77, *SE* = 0.02) invested a significantly higher proportion of their reproductive effort on the main inflorescence than perpetual accessions (mean = 0.29, *SE* = 0.01) or mutant genotypes (mean = 0.31, *SE* = 0.01; Figure 1f, Table 1; Supporting information Table S4). Moreover, we found no significant difference in the proportion of reproductive effort on the main stalk invested by perpetual accessions and mutant genotypes (Supporting information Table S4). However, despite the consistent differences in flowering behaviour between accession groups, there was considerable variation between the accessions and mutant genotypes within accession groups as well (Figure 1g).

**Table 1:** Statistical models describing the relationship between accession group and flowering and seed traits. Results shown are for statistical analyses of the greenhouse data set, including plants from greenhouse replicate experiments 1 and 2

Response variable	*df*	*F*	*p*	Partial *η* ^2^	Adjusted *R* ^2^
Proportion of RE on main inflorescence					
Model	5, 307	322.09	<0.001		0.84
Intercept	1	5,168.84	<0.001	0.94	
Accession group	2	741.79	<0.001	0.83	
Replicate	1	13.86	<0.001	0.04	
Accession Group*Replicate	2	13.44	<0.001	0.08	
Total number of flowers					
Model	5, 310	137.27	<0.001		0.68
Intercept	1	5,180.13	<0.001	0.94	
Accession group	2	49.06	<0.001	0.24	
Replicate	1	328.95	<0.001	0.52	
Accession Group*Replicate	2	9.30	<0.001	0.06	
DSDS50					
Model	5, 102	54.50	<0.001		0.72
Intercept	1	2,173.75	<0.001	0.96	
Accession Group	2	107.73	<0.001	0.69	
Replicate	1	5.99	<0.001	0.06	
Accession Group*Replicate	2	13.15	<0.001	0.22	
P50					
Model	5, 102	50.91	<0.001		0.71
Intercept	1	2,445.65	<0.001	0.96	
Accession Group	2	118.93	<0.001	0.72	
Replicate	1	1.26	0.27	0.01	
Accession Group*Replicate	2	0.68	0.51	0.01	
ABA sensitivity					
Model	3	41.49	<0.001		0.60
Intercept	1	730.885	<0.001	0.91	
ABA Concentration	1	101.75	<0.001	0.57	
Accession (Paj/*pep1‐1*/*pep1‐2*)	2	11.36	<0.001	0.23	
PBZ sensitivity					
Model	3	13.42	<0.001		0.34
Intercept	1	166.69	<0.001	0.70	
PBZ Concentration	1	37.76	<0.001	0.35	
Accession (Paj/*pep1‐1*/*pep1‐2*)	2	0.69	0.50	0.02	
GA sensitivity					
Model	3	7.47	<0.001		0.20
Intercept	1	2,045.86	<0.001	0.97	
GA Concentration	1	18.92	<0.001	0.20	
Accession (Paj/*pep1‐1*/*pep1‐2*)	2	1.54	0.22	0.04	

**Figure 2:**
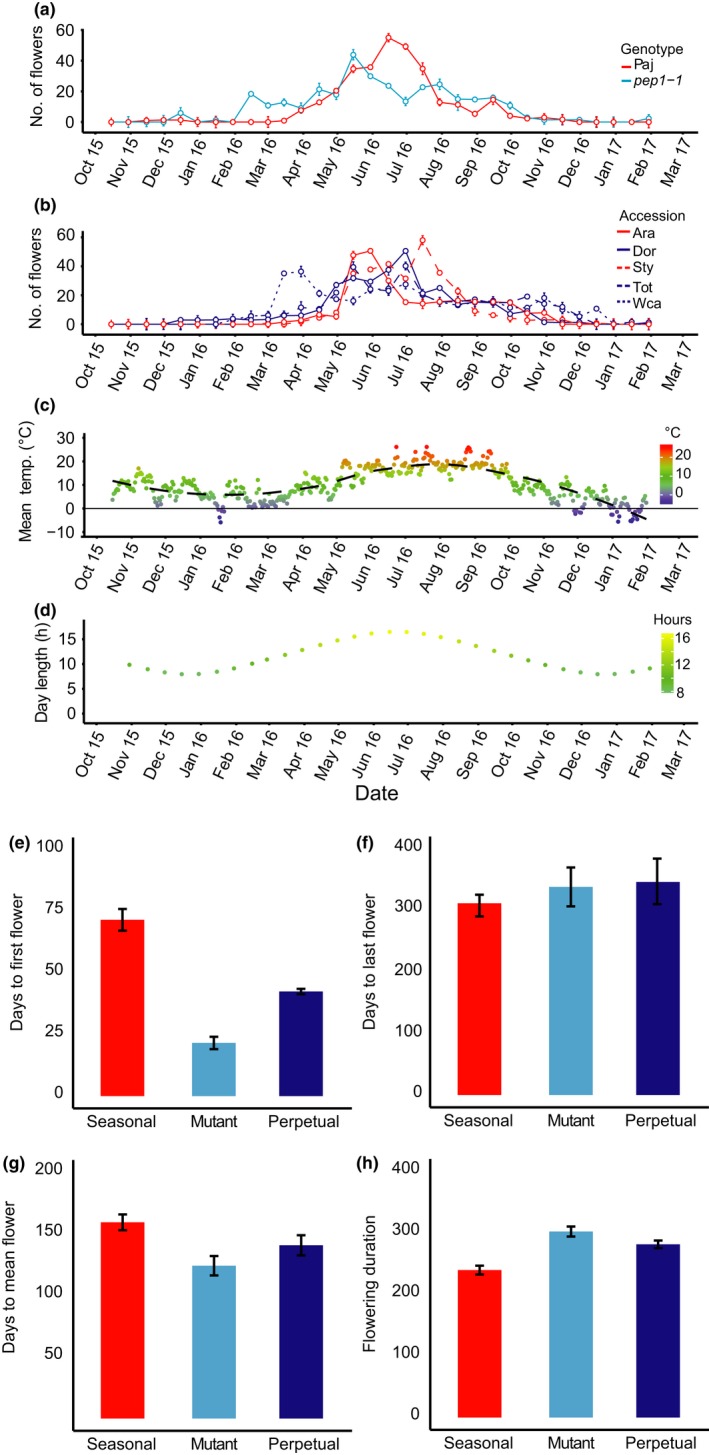
Perpetual flowering accessions and *pep1* mutants flowered earlier than seasonal flowering accessions and showed asynchronous flowering in the garden. (a, b) Flower production from October 2015 to February 2017 for (a) Pajares (Paj) and the *pep1‐1 *mutant; (b) seasonal and perpetual accessions. (c, d) Environmental conditions in the garden: (c) mean daily temperature. Colours show temperature and the dotted line shows a Loess curve fitted to weather data to visualize seasonal trends; (d) photoperiod (hours of sunlight per day) over time at the garden site. (e) A bar chart showing the mean number of days from 1 January to the onset of flowering by accession group; (f) a bar chart showing the mean number of days from 1 January to the end of flowering by accession group; (g) a bar chart showing the mean number of days from 1 January to the 50th percentile flower by accession group; (h) a bar chart showing the mean flowering duration (in days) by accession group; seasonal accessions (Paj, Ara and Sty) and the seasonal accession group are shown in red, perpetual accessions (Dor, Tot and Wca) and the perpetual accession group in blue, and mutants (*pep1‐1* and *pep1‐2*) and the mutant genotype group in cyan. Error bars indicate *SEM*. *N* = 16–23 per accession [Colour figure can be viewed at http://wileyonlinelibrary.com]

In the experimental garden, we also found differences in flowering schedules among accession groups (Figure 2a, b). Specifically, we found that *pep1‐1* plants displayed precocious flowering before the winter, resumed flowering by early February and maintained a high rate of flowering until 30 October (Figure 2a). In contrast, Paj plants began to flower by 30 March, reached peak flowering by mid‐June and completed flowering by 15 October. Statistical tests revealed that the mutant genotype group (mean = 384.9, *SE* = 18.7) produced a significantly higher number of flowers than the seasonal (mean = 295.7, *SE* = 12.1) or perpetual (mean = 330.9, *SE* = 16.6) accession groups (Table 2). However, we did not find a significant difference between seasonal and perpetual accession groups (Supporting information Table S4). As in the greenhouse, the seasonal accession group (mean = 0.81, *SE* = 0.02) invested a higher proportion of reproductive effort on the main inflorescence than the perpetual accession group (mean = 0.45, *SE* = 0.03) or the mutant group (mean = 0.43, *SE* = 0.02; Supporting information Table S4). No significant difference in the proportion of reproductive effort on the main inflorescence was detected between the perpetual and mutant groups (Supporting information Table S4).

**Table 2:** Statistical models describing the relationship between accession group and flowering and seed traits. Results shown are for statistical analyses of the experimental garden data set

Response variable	*df*	*F*	*p*	Partial *η* ^2^	Adjusted *R* ^2^
Proportion of RE on main inflorescence					
Model	2, 121	87.07	<0.001		0.79
Intercept	1	1,381.83	<0.001	0.97	
Accession group	2	87.07	<0.001	0.80	
Total number of flowers					
Model	2, 121	7.76	<0.001		0.23
Intercept	1	1,480.14	<0.001	0.97	
Accession group	2	7.76	0.001	0.27	
Date of onset of flowering					
Model	2, 121	279.24	<0.001		0.82
Intercept	1	1552.69	<0.001	0.93	
Accession group	2	279.24	<0.001	0.83	
Date of end of flowering					
Model	2, 121	7.68	0.001		0.10
Intercept	1	18,089.52	<0.001	0.99	
Accession group	2	7.68	0.001	0.12	
Date of mean flowering					
Model	2, 121	37.17	<0.001		0.38
Intercept	1	5,105.66	<0.001	0.98	
Accession group	2	37.17	<0.001	0.39	
Flowering duration					
Model	2, 121	140.18	<0.001		0.70
Intercept	1	13,762.37	<0.001	0.99	
Accession group	2	140.18	<0.001	0.70	
DSDS50					
Model	2, 46	65.06	<0.001		0.74
Intercept	1	786.24	<0.001	0.95	
Accession group	2	65.06	<0.001	0.75	
P50					
Model	2, 46	70.09	<0.001		0.75
Intercept	1	834.50	<0.001	0.95	
Accession group	2	70.09	<0.001	0.77	

Next, given the seasonal variation in temperature (Figure 2c) and day length (Figure 2d), we compared the date of onset of flowering, the date of the end of flowering, the duration of flowering and the date of mean flowering among accession groups (Figure 2e–h). Perpetual accessions (mean = 36.8 days after 1 January, *SE* = 1.7) and mutant genotypes (mean = 21.6, *SE* = 2.5) flowered significantly earlier than plants in the seasonal accession group (mean = 87.8, *SE* = 1.9; Figure 2e; Supporting information Table S4), but all accession groups ended flowering at roughly the same time late in the year (Figure 2f; Supporting information Table S4). However, we found significant differences in the date of mean flowering—that is the date by which 50% of all flowers had been produced (Figure 2g; Supporting information Table S4). Perpetual accessions (mean = 131.2 days after 1 January;* SE* = 2.7) and mutant genotype group plants (mean = 124.2; *SE* = 3.1) reached this date much earlier than seasonal accession plants did (mean = 161.1; *SE* = 3.2; Supporting information Table S4), presumably because they began to flower earlier. The dates of mean flowering for the perpetual accessions and mutants were before the warmest days of the 2016 summer (i.e., 30 June–15 August) and after the peak photoperiod at 21 June, while the date of mean flowering for the seasonal accessions occurred during this period. Consequently, we found that plants in the perpetual (mean = 286.0 days, *SE* = 3.7) and mutant accession groups (mean = 300.1, *SE* = 4.5) showed a significantly longer flowering duration than plants in the seasonal accession group (mean = 217.0, *SE* = 2.3; Figure 2h; Supporting information Table S4); however, we found no significant differences in flowering duration between the mutant and perpetual accession groups (Supporting information Table S4). Taken together, these results show that the presence of a functional *PEP1* allele is associated with profound differences in flowering behaviour, including a later onset of flowering, a later date of mean flowering and a shorter total duration of flowering.

**Figure 3:**
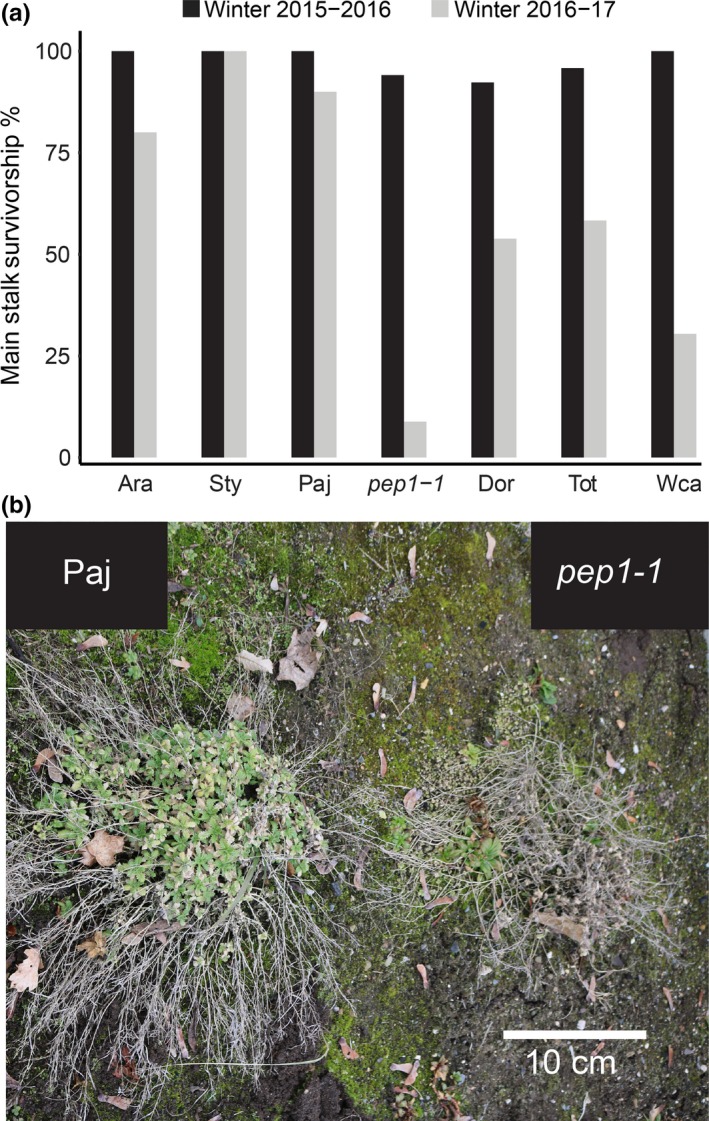
Perpetual flowering accessions and the *pep1‐1 *mutant have reduced survivorship in the garden. (a) Plant survivorship rate by accession after the 2015–2016 and 2016–2017 winters. (b) Representative Pajares and *pep1‐1* plants on 27 February 2017 [Colour figure can be viewed at http://wileyonlinelibrary.com]

We also found differences in survivorship between accessions. Plant mortality was assessed after the 2015–2016 and 2016–2017 winters, the second of which was much more severe than the first. Mortality scoring was simple—we examined plants at the end of the winter and considered only whether the plant that we originally planted in the garden was still alive or not, regardless of plant condition or whether offspring had already established themselves nearby. Interestingly, we found that plant persistence was compromised in perpetual accession and mutant groups, with these groups showing significantly lower survivorship than the seasonal accession group (Figure 3a; Supporting information Table S5); this was especially true after the more severe winter of the second year. Approximately 86% of Paj plants survived two winters, while only <10% of *pep1‐1* plants did (Figure 3a). After exposure to winter cold, the flowering axillary branches of Paj plants senesced, but the main stalk persisted; in contrast, most *pep1‐1* plants did not survive the winter (Figure 3b). However, even though most *pep1‐1* plants showed higher mortality, we observed that most plants showed the establishment of new seedlings from germinated seeds (Figure 3b) and/or clones from axillary branches rooted through adventitious roots (Supporting information Figure S2).

### 3.2 Perpetual flowering accessions and *pep1* mutants have reduced seed dormancy

We conducted germination trials to examine whether seeds produced by different accessions showed variation in the amount of after‐ripening required to release seed dormancy. The number of days of dry storage to reach 50% germination (DSDS50) was determined for each accession, with a higher DSDS50 value indicating higher seed dormancy.

**Figure 4:**
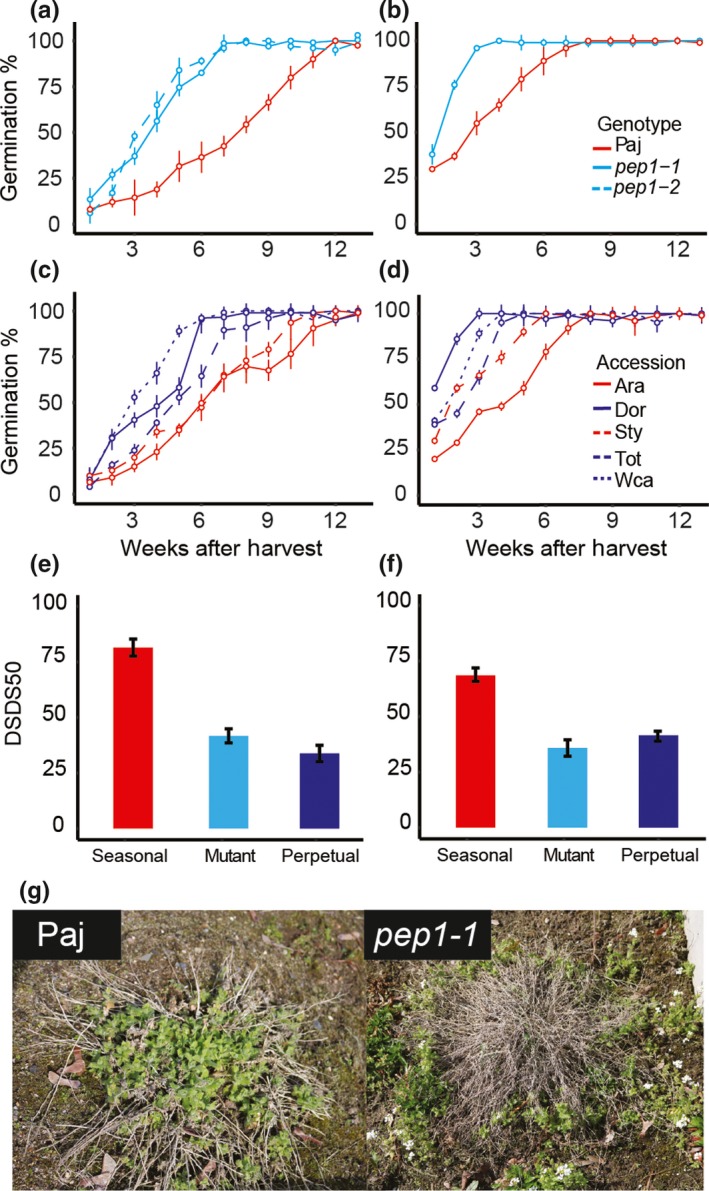
Perpetual flowering accessions and *pep1* mutants show reduced seed dormancy. Seed germination rates over 14 weeks of after‐ripening are shown for (a) Pajares (Paj), *pep1‐1* and *pep1‐2* plants grown in the greenhouse; (b) Paj and *pep1‐1 *plants grown in the garden; (c) seasonal and perpetual accessions grown in the greenhouse; (d) seasonal and perpetual accessions grown in the garden; (e) a bar chart showing DSDS50 by accession group for greenhouse plants; (f) a bar chart showing DSDS50 by accession group for garden plants; and (g) representative Paj and *pep1‐1* plants growing in the garden (24 March 2017). Seasonal accessions (Paj, Ara and Sty) and the seasonal accession group are shown in red, perpetual accessions (Dor, Tot and Wca) and the perpetual accession group in blue, and mutants (*pep1‐1* and *pep1‐2*) and the mutant genotype group in cyan. For a–d, data reported are the mean of six independent biological replicates for Paj and *pep1‐1*, or of four for all other accessions. Error bars indicate *SEM* [Colour figure can be viewed at http://wileyonlinelibrary.com]

In both the greenhouse and the garden, plants from the seasonal accession group showed higher seed dormancy than plants from the perpetual accession or mutant groups. In seeds matured in greenhouse plants, germination rates immediately after harvest were low for all genotypes. Seasonal accessions required 11–12 weeks of dry storage to reach 100% germination, while perpetual accessions and the *pep1* mutants reached 100% germination after only 6–9 weeks of dry storage (Figure 4a, c, e). The seasonal accession group showed higher DSDS50 values (mean = 53.4 days; *SE* = 1.9) than the perpetual accession group (mean = 27.3; *SE* = 1.1) or the mutant group (mean = 34.8; *SE* = 1.5; Supporting information Table S4). Pairwise comparisons between Paj and the *pep1‐1* and *pep1‐2* mutants also showed that Paj seeds (mean = 61.8; *SE* = 0.9) had higher DSDS50 values than either mutant (*pep1‐1 *mean = 37.8; *SE* = 1.1; *pep1‐2* mean = 25.1; *SE* = 1.5; Supporting information Table S3). Differences in DSDS50 were not influenced by temperature, since seeds from Paj and *pep1‐1* showed no difference when germinated at 10°C or 25°C (Supporting information Figure S3). Moreover, we found that the mean DSDS50 of the mutant genotype accession group was slightly higher than that of the perpetual accession group (Supporting information Table S4).

In the garden, germination rates upon harvest were considerably higher than in the greenhouse, although the trend among accession groups was similar (Figure 4a, b). Seasonal accessions reached near 100% germination after 6–8 weeks of after‐ripening, while perpetual accessions required 3–5 weeks of after‐ripening (Figure 4b, d, f). In the garden, nondormant seeds produced by perpetual accessions were often found to germinate shortly after being shed. Due to the relatively high mortality of *pep1‐1* and perpetual accession plants in the garden, it was not uncommon to find a cloud of small germinated seeds and small seedlings overgrowing a mother plant (Figure 4g). Statistical comparisons of the mean DSDS50 values of different accession groups revealed significant differences between the seasonal accession (mean = 61.3 days; *SE* = 2.8) and mutant groups (mean = 34.6; *SE* = 1.1) as well as between the seasonal accession group and the perpetual accession group (mean = 28.3; *SE* = 3.1; Supporting information Table S4). We also found no difference in mean DSDS50 between the mutant and perpetual accession groups (Supporting information Table S4). Comparisons between the DSDS50 values of the accession groups revealed greater differences in the garden compared to the greenhouse. Once again, we found significant differences in mean DSDS50 between Paj (mean = 35.7; *SE* = 1.4) and the two mutant genotypes (*pep1‐1* mean = 34.6; *SE* = 1.1; *pep1‐2 *mean = 44.0; *SE* = 1.7). Taken together, our results showed that the presence of a functional *PEP1* allele was consistently associated with higher DSDS50 values and, hence, higher seed dormancy.

### 3.3 Perpetual flowering accessions and *pep1* mutants have low seed longevity

To determine whether seeds produced by different accessions and mutants had altered seed longevity, we used both artificial and natural seed ageing tests. Controlled deterioration tests (CDTs) were used to assess seed survival in response to exposure to high humidity and high temperature. CDTs involve exposing nondormant seeds to stressful conditions for various intervals and assessing germination response afterwards. The half‐viability period (P50) is the interval (in days) required to reduce the germination rate to 50%. A higher P50 value is indicative of higher seed longevity.

**Figure 5:**
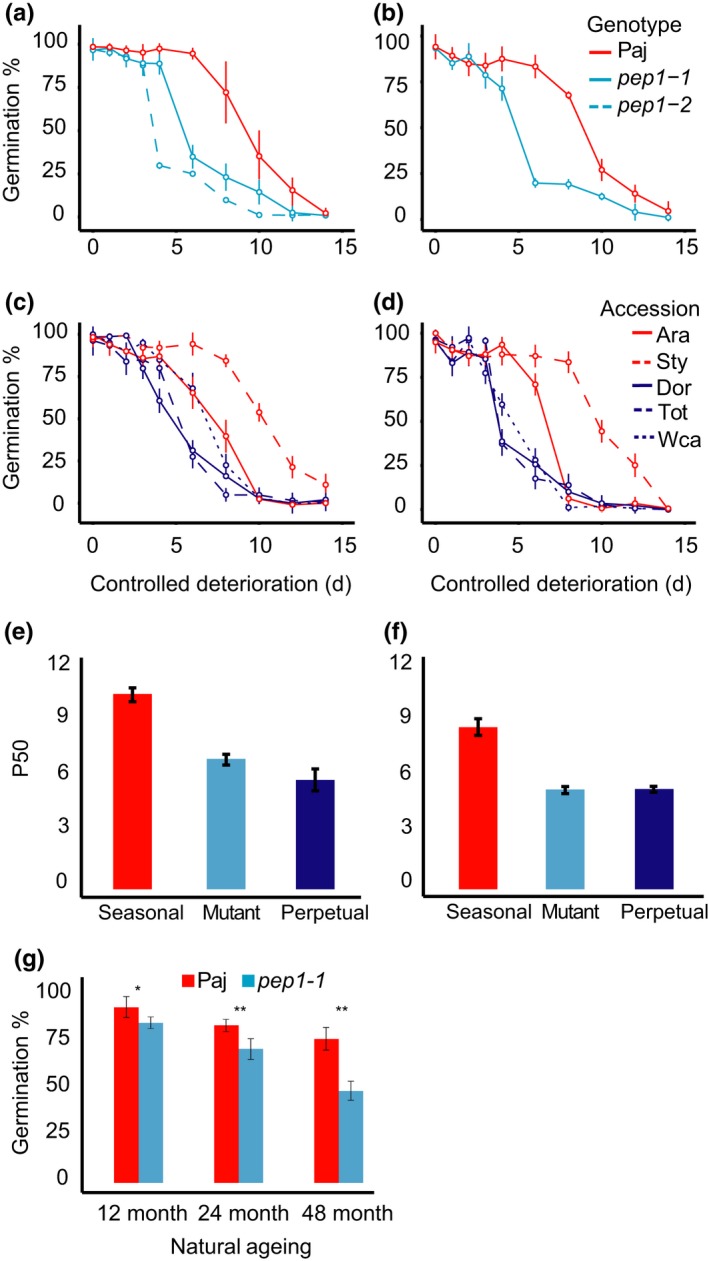
Perpetual flowering accessions and *pep1* mutants show reduced seed longevity. Mean germination rates of seeds after exposure to artificial ageing by a controlled deterioration test (CDT) from (a) Pajares (Paj), *pep1‐1* and *pep1‐2* plants grown in the greenhouse; (b) Paj and *pep1‐1 *mutant plants grown in the garden; (c) seasonal and perpetual accessions grown in the greenhouse; (d) seasonal and perpetual accessions grown in the garden; (e) a bar chart showing P50 by accession group for greenhouse plants; (f) a bar chart showing P50 by accession group for garden plants; and (g) a bar chart showing mean germination rates of *pep1‐1 *and Paj plants after natural ageing. Seasonal accessions (Paj, Ara and Sty) and the seasonal accession group are shown in red, perpetual accessions (Dor, Tot and Wca) and the perpetual accession group in blue, and mutants (*pep1‐1* and *pep1‐2*) and the mutant genotype group in cyan. For a–d, data reported are the mean of six independent biological replicates for Paj and *pep1‐1*, or of four for all other accessions. Error bars indicate *SEM*. Comparisons report Tukey HSD tests at **p < 0.01 (corrected for FDR) [Colour figure can be viewed at http://wileyonlinelibrary.com]

In all plants, we found that mean P50 significantly differed among accession groups in both the greenhouse and experimental garden populations (Figure 5a–d; Table 1; Supporting information Table S4). CDTs of seeds produced by plants in the greenhouse revealed that seasonal accession group seeds (mean = 8.6 days; *SE* = 0.3) showed significantly higher P50 values than seeds produced by plants from the mutant (mean = 4.7; *SE* = 0.2) or perpetual accession groups (mean = 5.0; *SE* = 0.1; Supporting information Table S4). No statistically significant differences were found in P50 between the mutant and perpetual accession groups (Supporting information Table S4). Pairwise comparisons of P50 values in Paj and mutant seeds revealed that the P50 values of seeds from Paj plants (mean = 9.2; *SE* = 0.1) were significantly higher than the P50 values of seeds from *pep1‐1* (mean = 5.0; *SE* = 0.1) or *pep1‐2* plants (mean = 3.6; *SE* = 0.1; Supporting information Table S3). In the experimental garden, we observed a similar pattern: seeds produced by plants from the seasonal accession group (mean = 8.5; *SE* = 0.3) showed significantly higher P50 values than seeds produced by plants from the mutant (mean = 4.8; *SE* = 0.2) or perpetual accession groups (mean = 4.7; *SE* = 0.1; Supporting information Table S4). We again found that the P50 values of seeds from Paj plants (mean = 8.8; *SE* = 0.3) were significantly higher than the P50 values of seeds from *pep1‐1* (mean = 4.8; *SE* = 0.2) or *pep1‐2* plants (mean = 6.8; *SE* = 0.2).

To corroborate the results of the controlled deterioration tests, we performed germination tests on Paj and *pep1‐1* seeds that had been naturally aged for one to four years. This test showed that Paj seeds showed significantly higher germinability than *pep1‐1* seeds after one, two and four years of natural ageing (Figure 5e; Supporting information Table S6). Thus, accession group was a strong predictor of P50 in all environments, with the presence of a functional *PEP1* being associated with higher P50 values and thus higher seed longevity.

### 3.4 *pep1 *mutants have reduced sensitivity to ABA and reduced expression of genes implicated in ABA signalling

**Figure 6:**
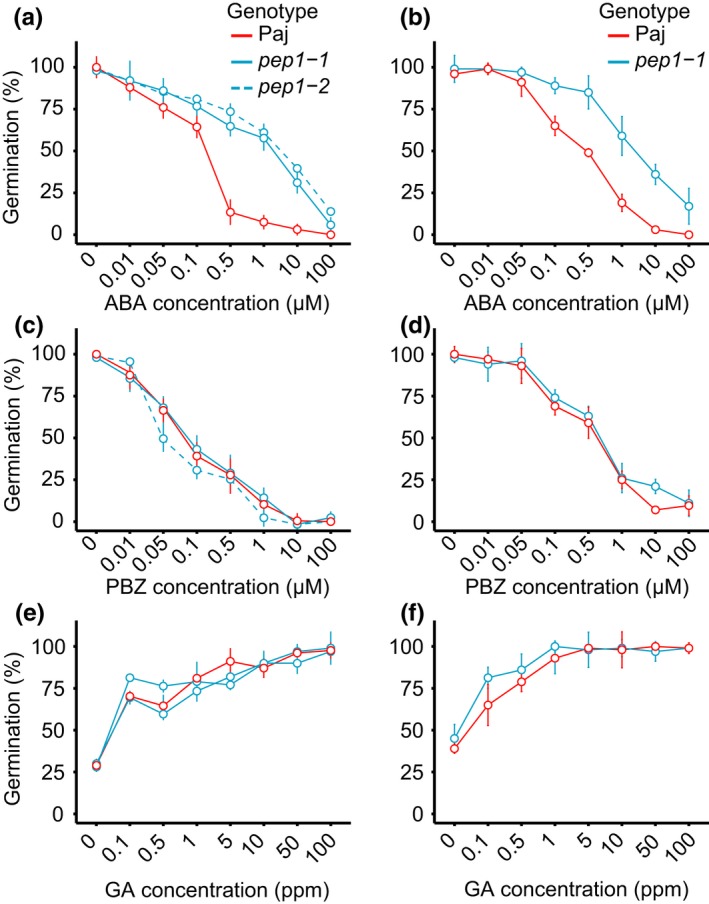
*pep1* mutants show higher sensitivity to exogenous ABA, but not exogenous GA or paclobutrazol. Seed sensitivity to ABA is shown for (a) greenhouse plants and (b) garden plants. Seed sensitivity to paclobutrazol is shown for (c) greenhouse plants and (d) garden plants. Seed sensitivity to GA is shown for (e) greenhouse plants and (f) garden plants. Pajares (Paj) is shown in red and *pep1* mutants in cyan. Data reported are the mean of six independent biological replicates for Paj and *pep1‐1*, and four for *pep1‐2*. Error bars indicate *SEM* [Colour figure can be viewed at http://wileyonlinelibrary.com]

To examine whether *PEP1* influences seed sensitivity to phytohormones, we conducted seed germination trials of Paj and *pep1* mutants in the presence of varying concentrations of ABA, GA and paclobutrazol (PBZ), an inhibitor that prevents endogenous GA biosynthesis. We found that Paj seeds were more sensitive to exogenous ABA than were *pep1‐1* and *pep1‐2* seeds, and the greatest difference in ABA sensitivity between accessions was between Paj and *pep1‐2* (Figure 6a, b; Supporting information Table S3). For example, the maximum difference between genotypes occurred at an ABA concentration of 0.5 μM, where Paj seeds (mean = 0.18; *SE* = 0.03) showed a much lower germination fraction than *pep1‐1* (mean = 0.66; *SE* = 0.05) or *pep1‐2* seeds (mean = 0.73; *SE* = 0.04). No differences in GA or PBZ sensitivity among seeds from different accessions were detected (Figure 6c–f; Supporting information Table S3).

### 3.5 Expression of genes implicated in ABA signalling in seeds is reduced in *pep1‐1*

**Figure 7:**
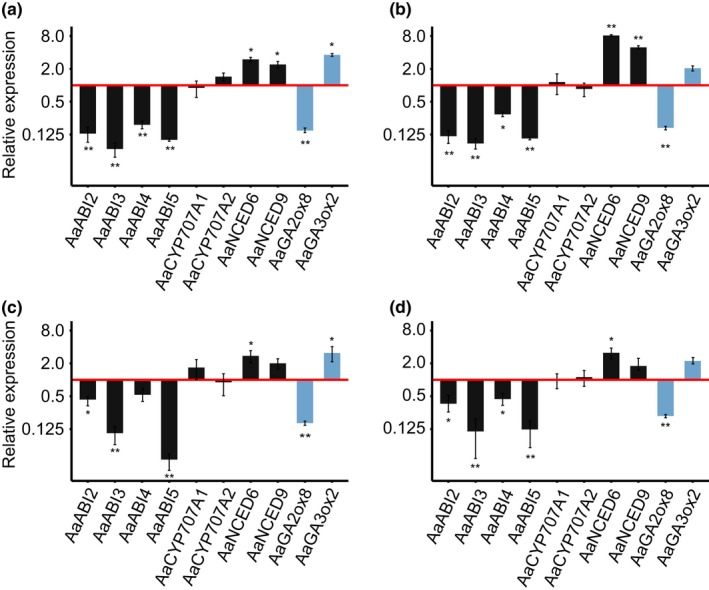
Genes involved in ABA signalling are differentially expressed between Pajares (Paj) and the *pep1‐1* mutant. Bars above/below the red line represent genes upregulated/downregulated in *pep1‐1* relative to Paj. Expression data are shown for (a) dry seeds from greenhouse plants; (b) dry seeds from garden plants; (c) 24 hr imbibed seeds from greenhouse plants; and (d) 24 hr imbibed seeds from garden plants. Bars represent means of three technical replicates each from four biological replicates. GA biosynthesis and signalling genes are shown in light blue, and ABA biosynthesis and signalling genes are shown in black. Error bars represent asymmetrical relative error (see Livak & Schmittgen, 2001). Comparisons report Tukey HSD tests at **p < 0.01 (corrected for FDR) [Colour figure can be viewed at http://wileyonlinelibrary.com]

We also compared the expression of genes that correlate with seed dormancy in *A. thaliana *in dry and imbibed *pep1‐1* and Paj seeds. Among genes tested, the ABA signalling genes *AaABI2*, *AaABI3* and *AaABI5* showed the largest differences between the two genotypes; each of these was more highly expressed in Paj compared to *pep1‐1* dry and 24 hr‐imbibed seeds (Figure 7a–d). In addition, the expression of *AaNCED6* and *AaNCED9* was higher in *pep1‐1* seeds than in Paj seeds. In *A. thaliana*, *NCED6* and *NCED9* are required for seed development—*nced6* and *nced9* knockout mutant plants produce less ABA and show reduced seed dormancy (Lefebvre et al., 2006). In addition, impaired expression of *ABI2*, *ABI3*, *ABI4* and *ABI5* in *A. thaliana* has been linked to ABA insensitivity and reduced seed dormancy (Finkelstein & Somerville, 1990; Kermode, 2005; Lopez‐Molina, Mongrand, McLachlin, Chait, & Chua, 2002). Our results also show that the expression of *AaGA2ox6* was upregulated (Supporting information Figure S4) and the expression of *AaGA2ox8* was downregulated in *pep1‐1* relative to Paj (Figure 7). The accumulation of GA_4_ and other bioactive gibberellins has long been known to be associated with reduced seed dormancy (Bewley, 1997; Hilhorst & Karssen, 1992). GA2‐oxidase (GA2ox) enzymes deactivate bioactive gibberellins (Yamaguchi, 2008). Although in our data the detected GA2ox enzymes show the opposite expression pattern between the two genotypes, the higher expression of GA2ox enzymes might be associated with increased seed dormancy.

## 4 DISCUSSION

### 4.1 *PEP1 *regulates multiple life history traits in the perennial life history of *A. alpina*

In temperate climates, many species—both annual and perennial—flower seasonally in the spring or early summer, concentrating flowering during favourable conditions. Perennials overwinter both as seeds and as mature plants, and to avoid wasting reproductive effort by flowering too late, the flowering episode in perennials is restricted. In *A. alpina*, *PEP1* contributes to perenniality in two ways—by preventing axillary branches from undergoing the floral transition and limiting flowering duration (Wang et al., 2009). In this study, we observed differences among genotypes with and without lesions in *PEP1* for each of these traits, both in plants reared in the greenhouse and in the garden. The intrinsic and environmental factors governing the decision to invest resources in a present reproductive episode instead of a future one (or vice versa) are complex, but previous work has shown that the primary resource trade‐off concerns vegetative development and reproduction (Lloyd, 1980).

We found that the perpetual accession and *pep1* mutant accession groups realized higher reproductive effort and emphasized perennial traits to a lesser degree than did genotypes in the seasonal accession group (which included Paj). Seasonal accessions in both greenhouse and garden environments invested a higher proportion of their reproductive effort on the main stalk compared to axillary branches. In contrast, most axillary branches in perpetual accessions flowered and produced seeds. In the garden, we also observed strong differences in plant mortality; perpetual genotypes (and the *pep1‐1* mutant) were less likely to survive periods of winter cold than seasonal accessions. We can therefore hypothesize that winter cold influences perpetual accessions to a greater degree than seasonal ones. This difference may be due to direct effects of PEP1, which has been shown to regulate cold response by binding to cold‐regulated genes (COR; Mateos et al., 2017) or by differences in intrinsic resource allocation schedules between seasonal and perpetual genotypes. Under greenhouse conditions, the *pep1‐1* mutant produced more flowers than Paj (Figure 1e; Supporting information Table S3) and did not complete flowering by the end of the experiment. Since we found no differences in the mean number of seeds per silique (Supporting information Figure S2), genotypes in the perpetual accession group are likely capable of producing many more seeds than genotypes in the seasonal accession group, at least in the first year.

A strategy involving high reproductive effort early in life at the cost of survival or future reproduction is characteristic of annuals. However, in contrast to the behaviour of perpetual accessions of *A. alpina*, annuals in predictable, seasonal environments die after reproduction and therefore experience strong selection to complete reproduction quickly. In addition, annuals experience strong selection to establish a multiyear seed bank by producing seeds that can remain dormant. In both the greenhouse and the experimental garden, the reproductive behaviour of perpetual accessions resembles a compromised perenniality rather than true annuality. However, our data were collected in an experimental garden, and the adaptive significance of this reproductive behaviour in natural environments is unknown. Future studies should incorporate field experiments (such as reciprocal transplants) designed to test the relative performance of seasonal and perpetual accessions in their native environments in terms of herbivore pressure, pollinator attraction, plant survival, intraspecific competition and other behaviours that may meaningfully impact fitness in the field. Moreover, the geographic distribution patterns of seasonal and perpetual accessions and the segregation of traits within and among populations should also be assessed by examining natural populations in the field. These experiments should focus on identifying the ecological and evolutionary factors that permit the maintenance of both seasonal and perpetual life history habits in natural environments.

### 4.2 The role of *PEP1 *on seed traits has diverged from *FLC*

In *A. thaliana*, there is strong evidence that the floral repressor *FLC* also affects seed traits. A study by Chiang et al. (2009) demonstrated that higher expression of *FLC* was linked to low dormancy and found that the effect of *FLC* appeared to be temperature‐dependent. That is, differences in germination rates were apparent only when seeds were germinated in cool (10°C) conditions. In addition, data from 52 accessions also revealed a positive relationship between *FLC* expression and seed germination at 10°C but not at 22°C. Consistent with this, mutants in autonomous‐pathway genes that ordinarily repress *FLC* (i.e., *FCA*, *FY* and *FPA*) show reduced germination (Auge, Blair, Karediya, & Donohue, 2017; Blair, Auge, & Donohue, 2017). However, the role of *FLC* in germination and dormancy can vary, possibly depending on experimental or environmental conditions. For instance, seeds from the *flc‐101* mutant and those produced by Col‐0 wild‐type plants showed similar dormancy levels (Liu et al., 2011). Furthermore, Chen et al. (2014) found that seeds from the *flc‐21* mutant showed lower dormancy than did those from the L*er *wild‐type background. This was observed when seeds were germinated both at 22 and 10°C. Blair et al. (2017) argued that since the temperature range facilitating seed germination itself depends on dormancy, the temperature at which *FLC* regulates germination may also vary with dormancy. In this study, our results indicate that the role of *PEP1* on seed traits in *A. alpina *might have diverged from the role of *FLC*. Seasonal flowering accessions that have a functional *PEP1* allele consistently show higher seed dormancy compared to accessions and mutants that have lesions in *PEP1*. In addition, we found no evidence that the effect of *PEP1* on seed traits was temperature‐dependent. At 23°C, we found consistent differences in both seed dormancy and longevity between *pep1* mutants and Paj, as well as between the perpetual and seasonal accession groups and the mutant and seasonal accession groups (Figure 4; Supporting information Tables S3 and S4). These differences were consistent regardless of whether seed maturation occurred in the greenhouse or the garden (Figures 4, 5). In addition, while we found differences in germination fraction between *pep1‐1* (mean germination fraction = 0.20; *SE* = 0.06) and Paj seeds (mean = 0.17; *SE* = 0.04) germinated at 10°C, the magnitude of these differences was no greater than those at 23°C (Supporting information Figure S3, Table S7).

Seasonal flowering accessions also consistently showed higher seed longevity. These results differ from previous studies of *A. thaliana*, where seeds with high seed dormancy were found to have low seed longevity (Clerkx, Blankestijn‐De Vries et al., 2004). Furthermore, while in general seeds produced by garden plants consistently showed reduced seed dormancy relative to seeds produced by greenhouse plants, the differences in seed dormancy and longevity between Paj and *pep1* mutants—and between seasonal and perpetual accessions overall—were clear in all cases, suggesting a genetic contribution to these differences. In *A. thaliana*, differences in seed longevity between *FLC *mutants have not yet been systematically studied. However, Clerkx, El‐Lithy et al. (2004) performed controlled deterioration and natural ageing tests on seeds from the accessions Landsberg erecta (L*er*) and Columbia (Col), which have a nonactive and an active *FLC* allele, respectively (Michaels, He, Scortecci, & Amasino, 2003). Both tests revealed that Col had significantly higher seed longevity than L*er*. This suggests that *FLC* expression is associated with higher seed longevity, which agrees with the results we report here for *A. alpina*. However, L*er *and Col‐0 plants also have other genetic differences which may contribute to differences in seed longevity. Natural variation studies have identified QTLs that map to *FLC* that are also associated with traits that have been linked to variation in seed longevity, such as circadian period length and water use efficiency (Clerkx, El‐Lithy et al., 2004; McKay et al., 2003; Swarup et al., 1999). In general, the mechanism by which *FLC* may affect seed longevity in *A. thaliana* is unknown and requires further investigation.

The differences in gene regulation between Paj and *pep1‐1* differ from the ones reported in studies of *FLC* and seed dormancy. In *A. thaliana*, *FLC* expression is positively correlated with expression of the ABA catabolism gene *CYP707A2*, as well as the GA biosynthesis genes *GA20ox1* and *GA3ox1* (Chiang et al., 2009). In general, the actions of these genes increase the concentration of bioavailable GA and reduce the concentration of ABA, and Chiang et al. (2009) speculated that *FLC* was associated with lower seed dormancy due to altered regulation of GA biosynthesis or signalling. In *A. alpina,* we did not find differences in sensitivity to exogenous paclobutrazol or GA between *pep1‐1* and Paj seeds, whereas we did find differences in sensitivity to exogenous ABA. We also found no differences in *CYP707A2*, *GA20ox1* or *GA3ox1 *expression between Paj and the *pep1‐1* mutant. Instead, *GA2ox6 *and *GA3ox2* were upregulated and *GA2ox8* was downregulated in *pep1‐1* relative to Paj (Figure 7; Supporting information Figure S4). In a recent ChIP‐seq comparison of the targets of FLC and PEP1 in leaves and apices in *A. alpina*, Mateos et al. (2017) reported that *AaGA3ox2* and *AaGA2ox8* were direct targets of PEP1, but the corresponding orthologs were not direct targets of FLC. In addition, this study also reported that *AaABI5* was a direct target of PEP1, but that *ABI5* was not a direct target of FLC. Here, we found that genes involved in ABA sensitivity—including *NCED6, NCED9, ABI2*, *ABI3*, *ABI4* and *ABI5—*were differentially expressed between Paj and *pep1* seeds. Two MADS‐box transcription factors similar to *PEP1*—*AGAMOUS‐LIKE‐21* (*AGL21*) and *AGL67—*affect seed traits in *A. thaliana, and AGL67 *in particular is seed‐specific and has also been linked to altered ABA signalling (Bassel et al., 2011). Because ABA and GA biosynthesis and signalling are regulated by complex networks of feedback regulation, we can hypothesize that differential expression of GA‐related genes is due to knock‐on effects from differential expression of ABA‐related genes or vice versa.

Taken together, our study highlights the functional divergence between the role of *FLC* and *PEP1*. In *A. thaliana*, *FLC* promotes seed germination, whereas in *A. alpina* *PEP1* represses seed germination. To a lesser extent, there is evidence of functional divergence of *FLC* and *PEP1 *with respect to seed longevity. Previous studies in *A. thaliana* have demonstrated that higher seed dormancy is associated with lower seed longevity (Nguyen and Bentsink (2015), Rajjou & Debeaujon, 2008, Shen et al., 2018). However, other studies have shown that there is no relationship between these traits (Debeaujon, Leon‐Kloosterziel, & Koornneef, 2000; Thompson, Ceriani, Bakker, & Bekker, 2003). In our study, we demonstrated a positive relationship between seed dormancy and longevity which is dependent on *PEP1*.

The functional significance of this divergence may be related to life history differences between *A. thaliana* and *A. alpina*, the most basic of which is the fact that *A. thaliana* is an annual and *A. alpina* is a perennial. Given that for temperate‐biome species the dominant seasonal cue regulating seed germination is temperature, the optimal seed dormancy levels for *A. thaliana *are likely different from the levels required for *A. alpina*, and the functional divergence of *FLC*/*PEP1* may be an adaptation to these differences. Specifically, the fact that strong *FLC* alleles and low seed dormancy are both more common at high latitudes is evidence that low seed dormancy in *A. thaliana* facilitates early seedling establishment—and consequently the development of a winter annual life history—in northern accessions of *A. thaliana* (Atwell et al., 2010; Shindo, Lister, Crevillen, Nordborg, & Dean, 2006). In contrast, in *A. alpina* strong *PEP1* alleles are associated with high seed dormancy and may facilitate spring germination by preventing germination during the winter, whereas seedling establishment of perpetual accessions—which show lower winter survival, earlier flowering and lower seed dormancy—may occur in the late summer or the autumn. Although our study provides limited support for this explanation (i.e., we find consistent differences among accessions in a controlled environment), additional experiments designed to assess the relative fitness of seasonal and perpetual accessions in the field should be conducted to test these hypotheses in a natural environment. In addition, differences in seed longevity may permit seasonal accessions to distribute reproductive risk over a longer period of time—that is among years by mother plants versus by dormant seeds.

In *A. thaliana,* there is evidence that intraspecific variation at even a single locus may result in substantial life history changes. For example, the bHLH transcription factor *SPATULA* (*SPT*) controls the germination response to cold and light by integrating light and temperature signalling in the seed (Penfield et al., 2005). Interestingly, there is evidence that *SPT* alleles vary among *A. thaliana* accessions, thereby giving rise to a range of possible seed responses to chilling; this in turn can facilitate the production of a variety of life history strategies, including extreme summer annual and winter annual strategies (Penfield & Springthorpe, 2012; Springthorpe & Penfield, 2015). In general, *PEP1* may play a similar role in natural populations of *A. alpina*—that is variation at the *PEP1* locus may be associated with adaptive variation in life history strategies in *A. alpina*. Thus, despite the fact that *PEP1* and *FLC* may be functionally divergent, these genes may play analogous roles in regulating life history adaptation in *A. alpina* and *A. thaliana*, respectively. However, to determine whether or not the perpetual life history strategy (e.g., compromised perenniality) is adaptive or not will require further study of *A. alpina* accessions in the field. In particular, the adaptive significance of the perpetual habit should be tested by new experimental manipulations (e.g., reciprocal transplants) of natural accessions differing in *PEP1* function in their natural habitats.

## 5 CONCLUSION AND OUTLOOK

Identifying the conditions in which an annual life history can obtain a demographic advantage over a perennial one has been a major project in life history theory (Hughes, 2017; Hughes & Simons, 2014). However, few models explicitly consider the role played by genetic elements in the regulation of life history traits, or the degree to which trade‐offs can be constrained by pleiotropy. In *A. alpina*, previous studies have shown that genotypes with lesions in *PEP1 *show compromised perenniality and continuous flowering.

However, in this work we report that these genotypes—as well as the *pep1* mutants—also show reduced seed dormancy and longevity. Thus, *PEP1* regulates multiple stages of the *A. alpina* life cycle and facilitates perenniality both by limiting reproductive allocation and by preventing precocious seed germination. In the controlled environment in which we compared the relative fecundity and mortality of different natural accessions of *A. alpina*, perpetual accessions invested more in seed production but produced seeds that germinated quickly. Moreover, these accessions appeared to compensate for high winter mortality by producing more seedlings. We speculate that since fitness optima vary among years and environments, the pleiotropic effects of flowering time regulators such as *PEP1* may prevent the simple, independent optimization of life history strategies. However, the adaptive significance of PEP1’s pleiotropic effect on flowering and seed traits in natural environments should be assessed by a field experiment where measures of lifetime fitness can be directly compared.

Pleiotropic regulation of different major developmental transitions affecting different life history stages is not predicted by theoretical life history models, which generally consider the optimization of the timing of such transitions to be distinct questions, and therefore assume that they can be optimized independently (Young, 1981). For instance, annual‐semelparous and perennial‐iteroparous life history strategies are thought to be adaptations to divergent adult and juvenile mortality rates. However, where pleiotropic regulation prevents the independent optimization of traits contributing to adult and juvenile fitness, the assumption that this trade‐off exists may be too simplistic. In annual species, differences in the timing of the initiation of flowering have been noted to have important knock‐on effects on the time in which seeds are produced, and hence on the optimal degree of dormancy that seeds should have. Thus, life history models of *Arabidopsis* have suggested that flowering time optimization may constrain the range of available seed environments and sustain life history strategies (Springthorpe & Penfield, 2015). Perennial species face a complex set of trade‐offs that annuals do not face because they must invest in growth and reproduction in successive years. However, models of optimal perennial reproduction emphasize the sensitivity of evolutionarily stable strategies for perennials to the values of age‐specific traits including juvenile and adult survivorship, reproductive rates and growth rates (Iwasa & Cohen, 1989; Kozlowski & Wiegert, 1987; Pugliese & Kozlowski, 1990; Wang, Li, & Wang, 2016). Consequently, genetic or developmental constraints—such as pleiotropy—that prevent the independent optimization of vegetative and reproductive traits may have even more profound evolutionary implications for perennials than for annuals. Thus, understanding the nuanced role of major hub regulators such as *PEP1* on fitness traits at multiple life stages may be specifically important for understanding natural variation in life history strategies in wild populations of perennial species.

## AUTHOR CONTRIBUTIONS

P.W.H., W.J.J.S. and M.C.A. designed the study. P.W.H. performed the research. P.W.H., W.J.J.S. and M.C.A. analysed the data. P.W.H., W.J.J.S. and M.C.A. wrote the paper.

## Data Availability

Data in this study have been deposited on line and are available from the Dryad Database, https://doi.org/10.5061/dryad.r4599c5.
