# Supplementary material for: Seed traits are pleiotropically regulated by the flowering time gene PERPETUAL FLOWERING 1 (PEP1) in the perennial Arabis alpina
Source: Mol Ecol. 2019 Mar 15;28(5):1183–201. doi: 10.1111/mec.15034 (PMC6850658; doi:10.1111/mec.15034)
Supplement: Supplementary file 1 [file MEC-28-1183-s001.docx]

## Supporting Information

**Article title:**

Seed traits are pleiotropically regulated by the flowering time gene *PERPETUAL FLOWERING 1* (*PEP1*) in the perennial *Arabis alpina*

**Authors:**

P. William Hughes, Wim J.J. Soppe, Maria C. Albani

The following Supporting Information is available for this article:

**
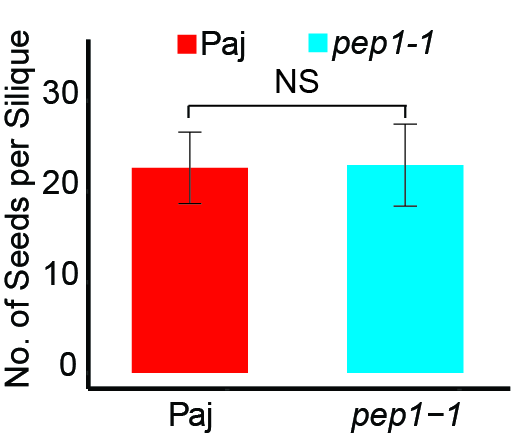
**

**Figure S1.** The number of seeds per silique does not significantly differ between Pajares (Paj) and *pep1-1* plants. Bars show mean total number of seeds per silique, computed from five technical replicates each of six biological replicates. Error bars indicate SEM. Paj is shown in red and *pep1-1* in cyan.

**
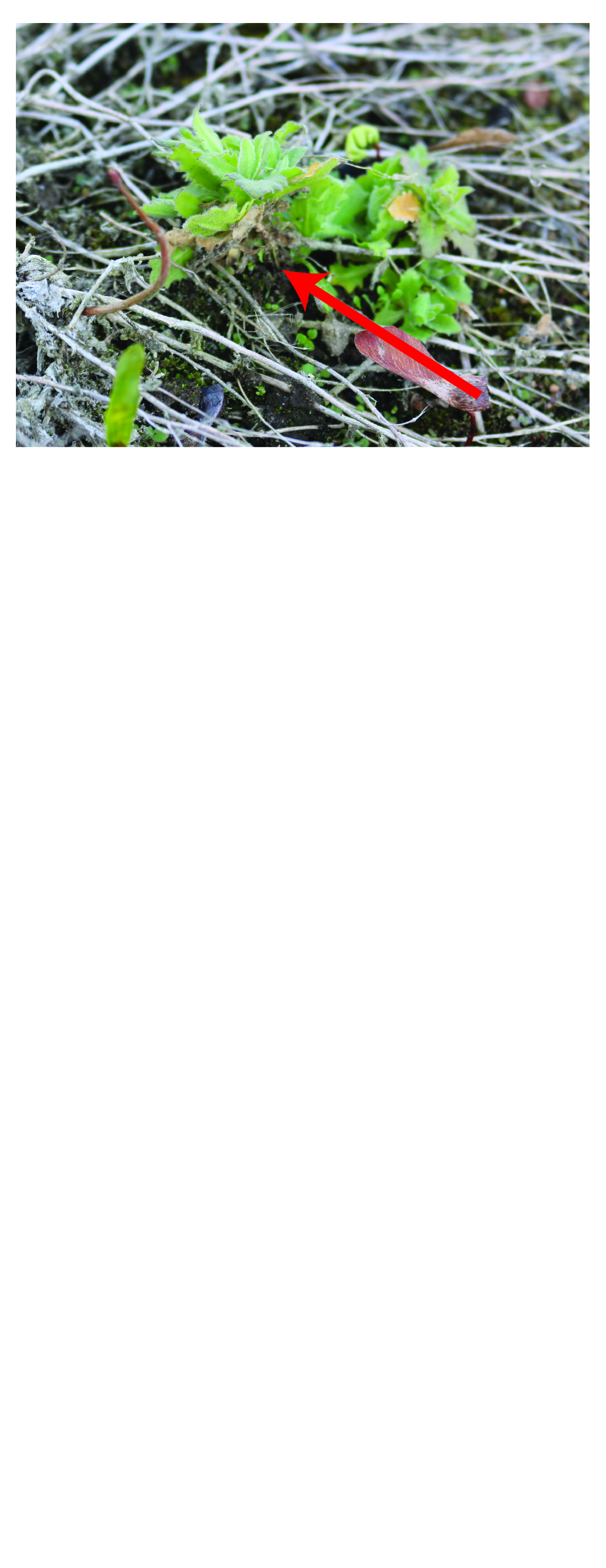
**

**Figure S2.**  *pep1-1* plant sprouting from an adventitious root in the experimental garden.

**
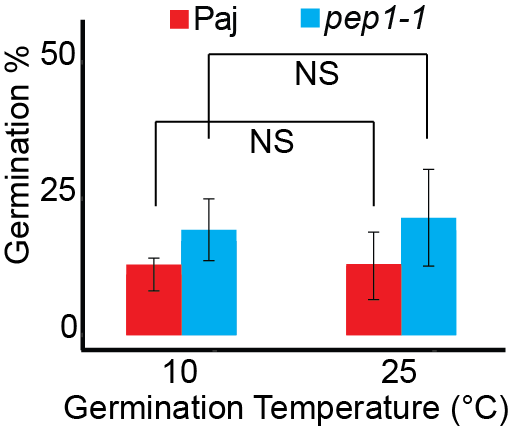
**

**Figure S3.** Relative differences in the germination rates of *pep1-1* and Pajares (Paj) seeds do not depend on germination temperature. Bars show mean germination fraction of three technical replicates each from four biological replicates. Seeds were obtained from Greenhouse Replicate Experiment 1. Error bars indicate SEM. Paj is shown in red and *pep1-1* in cyan.

**
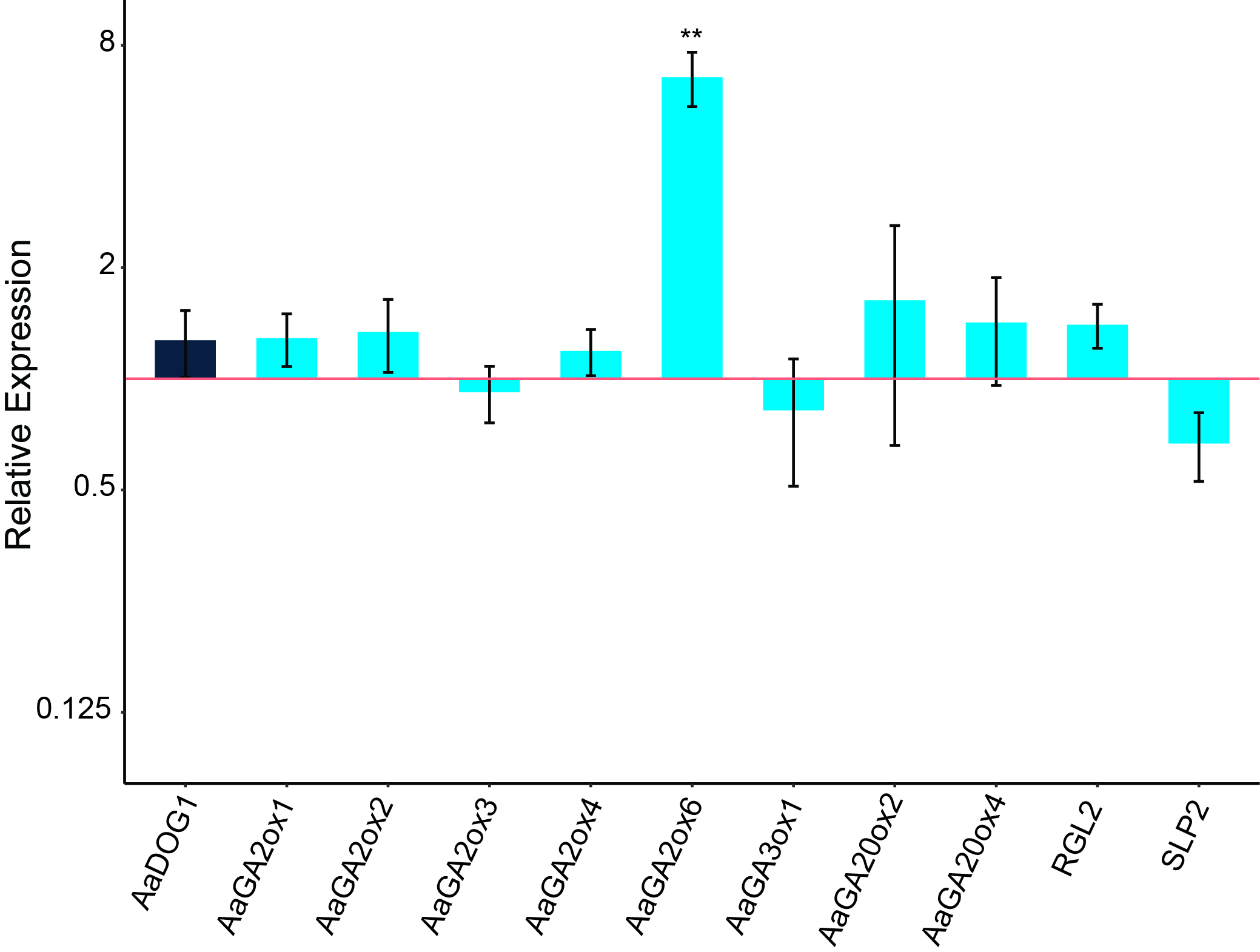
**

**Figure S4.** RT-qPCR data shows that one gene (*AaGA2ox6*) is differentially expressed between Pajares (Paj) and the *pep1-1* mutant, but many others involved in GA biosynthesis and catabolism are not. Bars above/below the red line represent genes upregulated/downregulated in *pep1-1* relative to Paj. Data shown is expression data for dry seeds. Bars represent mean values of three technical replicates each of four biological replicates. Genes associated with gibberellic acid biosynthesis and signaling are shown in light blue, and *DOG1* is not directly involved in hormone biosynthesis, and is shown in dark blue. Error bars represent asymmetrical relative error (determined as per Livak and Schmittgen (2001)). Comparisons report Tukey HSD tests showing significant differences at **P*<0.05 or ***P*<0.01 (corrected for FDR).

**SUPPLEMENTAL TABLES**

**Table S1.** qRT-PCR primer sequences used in this study.

| Gene | Primer | Sequence (5' to 3') |
| --- | --- | --- |
| AaKU70 | F | CTGATTTGGTGCCAAGTTTGT |
|  | R | CCTCATACTCCCAAAAGGATCA |
| AaWD40 | F | GCTTCACCTTTTCGCAAATC |
|  | R | CAGAGAGGAGCGATCCAAAC |
| AaABI2 | F | TTCAGAATCTCTAAATGGCTGAAAC |
|  | R | AAAAGGACTAAAATCAGAGTTTCCTG |
| AaABI3 | F | ATGAAAACCTTGCATGTGGC |
|  | R | ATCAGCTTCAAAAGCTGTTTCAT |
| AaABI4 | F | GAGATCCGAGAGCCACGTAA |
|  | R | GGAAGAGGAGGTGGAAGGAG |
| AaABI5 | F | GCGAAACAGCGGGTATAACA |
|  | R | TTCCAGCTCCGCTTTGTAGG |
| AaCYP707A1 | F | atggttttgtactttcagatggt |
|  | R | cacatcgatctccgacttcc |
| AaCYP707A2 | F | ATCCATCACTCTCCGGAATTCTTCC |
|  | R | TCCATTCCCAAATGGCATGAACG |
| AaNCED6 | F | CATAATCACCCACTTCCT |
|  | R | GGAG CGAA ATTA CCTG AT |
| AaNCED9 | F | GGGGTCGGCTGTTTTAGGAA |
|  | R | GGAAAACGCCATGATCTCACA |
| AaDOG1 | F | GATGGCCGAGTGAAACC |
|  | R | AACTGGCCATAGCTTCTTC |
| AaGA2ox1 | F | ATCAATGGCGGTATTGTCTAAAC |
|  | R | ACCTTGAAGAAGCCGAAGTC |
| AaGA2ox2 | F | GCAAGAAGCTATGGGTTT |
|  | R | GTATATTCTTTTAAATGCTAATCCTCAA |
| AaGA2ox3 | F | AGAAGGAACAAGAGATGGGATTCGG |
|  | R | CAGTACTGAGATCAAACGAAACAGAGAGT |
| AaGA2ox4 | F | AGTGTAAGGCATAGAGCATTGAC |
|  | R | GATGAATCAGCCACGGTTGTA |
| AaGA2ox6 | F | CGTTAAGCGGCGTTA |
|  | R | CAAGCCGTCACGTGAGC |
| AaGA2ox8 | F | ACCAAAAAGATGATATGTGGACGT |
|  | R | TCTAGAGTGGGTCCATGAGCT |
| AaGA3ox1 | F | AAGCACGCTTTTGAACAAACCA |
|  | R | GGTCTAGCCGCCCATACC |
| AaGA3ox2 | F | GGGTCGAGTCGGTATGA |
|  | R | TGGTCCGAAGGTTTCAC |
| AaGA20ox2 | F | TAACGTGAGATCTGGTGTTTGG |
|  | R | GAACAATTTGGGTACGTGTCTT |
| AaGA20ox4 | F | GTCCTGTCCCAAGTGCAACATCC |
|  | R | GTCTTGGCATCGACAGGAACCAT |
| AaRGL2 | F | TCAAGCCGGAGCTATG |
|  | R | AATGCATCTGTAAAACCTCT |
| AaSLP2 | F | ATCTTCTTACCACAAGACAC |
|  | R | ATCGGTCAGACGAATCGA |

**Table S2.** Gene IDs and accession numbers of genes studied. Accession numbers refer to the GenBank database (https://www.ncbi.nlm.nih.gov/genbank/).

| Gene | Gene ID | GenBank Accession Number | Gene ID of *A. thaliana* Ortholog(s) | Accession Number for *A. thaliana* Ortholog(s) |
| --- | --- | --- | --- | --- |
| PEP1 | Aa_G579940 | JX310558 | AT5G10140 | NM_121052 |
| AaSLP2 | Aa_G180420 | KFK28394 | AT4G34980 | NM_101705 |
| AaRGL2 | Aa_G47610 | KFK38060 | AT3G03450 | NM_111216 |
| AaGA20ox4 |  | LT669789 (6309886-6310203) | AT1G60980 | NM_104778 |
| AaGA20ox2 | Aa_G598100 | KFK26790 | AT5G51810 | NM_124560 |
| AaGA3ox2 | Aa_G312140 | LT669789 (36203501-36204074) | AT1G80340 | NM_106683 |
| AaGA3ox1 | Aa_G112460 | LT669788 (13798617- 13799201) | AT1G15550 | NM_101424 |
| AaGA2ox8 | Aa_G37650 | KFK28698 | AT4G21200 | NM_118239 |
| AaGA2ox6 | Aa_G6340 | KFK42605 | AT1G02400 | NM_100121 |
| AaGA2ox4 | Aa_G418770 | LT669788 (872938-873537) | AT1G47990 | NM_103695 |
| AaGA2ox3 | Aa_G291640 | KFK36353 | AT2G34555 | NM_129007 |
| AaGA2ox2 | Aa_G655020 | KFK44827 | AT1G30040 | NM_102743 |
| AaGA2ox1 | Aa_G470650 | KFK42227 | AT1G78440 | NM_106491 |
| AaDOG1 | Aa_G419960 | KFK28541 | AT5G45830 | NM_001344641 |
| AaNCED9 | Aa_G470690 | LT669789 (35493039- 35494745) | AT1G78390 | NM_106486 |
| AaNCED6 |  | LT669790 (18909779-18911531) | AT3G24220 | NM_113327 |
| AaCYP707A1 | Aa_G357050 | KFK39283 | AT4G19230 | NM_202845 |
| AaCYP707A2 | Aa_G425700 | KFK32241 | AT2G29090.1, AT2G29090.2 | NM_128466 |
| AaABI5 | Aa_G282200 | LT669791 (40901657- 40902777) | AT2G36270 | NM_129185 |
| AaABI4 | Aa_G115390 | LT669791 (47064682- 47065535) | AT2G40220 | NM_129580 |
| AaABI3 | Aa_G385870 | LT669790 (17142034- 17143722) | AT3G24650 | NM_113376 |
| AaABI2 | Aa_G168080 | LT669795 (38730621-38731242) | AT5G57050 | NM_125087 |
| AaKU |  | KFK43907 | AT1G16970 | NM_101558 |
| AaWD |  | LT669791 (50100562-50101716) | AT2G43770 | AY093182 |

**Table S3.** Pairwise comparisons between the Paj, *pep1-1*, and *pep1-2* genotypes. Results shown are for the greenhouse dataset, including plants from Greenhouse Replicate Experiments 1 and 2.

| **Variable** | **Pairwise Comparison** | **Estimate** | **SE** | **p** |
| --- | --- | --- | --- | --- |
| Proportion of RE on Main Inflorescence | Paj-*pep1-1* | 0.46 | 0.02 | <0.001 |
|  | Paj-*pep1-2* | 0.42 | 0.02 | <0.001 |
|  | *pep1-1-pep1-2* | -0.04 | 0.02 | 0.15 |
| Total Number of Flowers | Paj-*pep1-1* | -71.90 | 7.54 | <0.001 |
|  | Paj-*pep1-2* | -88.81 | 8.67 | <0.001 |
|  | *pep1-1-pep1-2* | -16.92 | 8.67 | 0.16 |
| DSDS50 | Paj-*pep1-1* | 25.39 | 1.32 | <0.001 |
|  | Paj-*pep1-2* | 37.29 | 1.81 | <0.001 |
|  | *pep1-1-pep1-2* | 11.90 | 1.80 | <0.001 |
| P50 | Paj-*pep1-1* | 4.18 | 0.15 | <0.001 |
|  | Paj-*pep1-2* | 5.57 | 0.21 | <0.001 |
|  | *pep1-1-pep1-2* | 1.40 | 0.20 | <0.001 |
| ABA Sensitivity | Paj-*pep1-1* | -0.12 | 0.05 | 0.03 |
|  | Paj-*pep1-2* | -0.23 | 0.04 | <0.001 |
|  | *pep1-1-pep1-2* | -0.11 | 0.05 | 0.07 |
| Paclobutrazol Sensitivity | Paj-*pep1-1* | -0.05 | 0.08 | 1.00 |
|  | Paj-*pep1-2* | -0.09 | 0.08 | 0.73 |
|  | *pep1-1-pep1-2* | -0.04 | 0.07 | 1.00 |
| GA Sensitivity | Paj-*pep1-1* | 0.05 | 0.04 | 0.52 |
|  | Paj-*pep1-2* | 0.06 | 0.04 | 0.32 |
|  | *pep1-1-pep1-2* | 0.01 | 0.04 | 1.00 |

**Table S4.** Pairwise comparisons between Accession Groups.

| **Variable** | **Environment** | **Pairwise Comparison** | **Estimate** | **SE** | **p** |
| --- | --- | --- | --- | --- | --- |
| Proportion of RE on Main Inflorescence | Greenhouse | Seasonal-Perpetual | 0.47 | 0.01 | <0.001 |
|  |  | Seasonal-Mutant | 0.46 | 0.02 | <0.001 |
|  |  | Perpetual-Mutant | -0.01 | 0.02 | 1.00 |
| Total Number of Flowers | Greenhouse | Seasonal-Perpetual | -23.19 | 5.49 | <0.001 |
|  |  | Seasonal-Mutant | -71.48 | 7.22 | <0.001 |
|  |  | Perpetual-Mutant | -48.29 | 7.04 | <0.001 |
| DSDS50 | Greenhouse | Seasonal-Perpetual | 26.03 | 1.84 | <0.001 |
|  |  | Seasonal-Mutant | 20.19 | 2.16 | <0.001 |
|  |  | Perpetual-Mutant | -5.84 | 2.18 | 0.03 |
| P50 | Greenhouse | Seasonal-Perpetual | 3.59 | 0.27 | <0.001 |
|  |  | Seasonal-Mutant | 3.92 | 0.31 | <0.001 |
|  |  | Perpetual-Mutant | 0.33 | 0.32 | 0.88 |
| Proportion of RE on Main Inflorescence | Garden | Seasonal-Perpetual | 0.37 | 0.03 | <0.001 |
|  |  | Seasonal-Mutant | 0.38 | 0.04 | <0.001 |
|  |  | Perpetual-Mutant | 0.01 | 0.04 | 1.00 |
| Total Number of Flowers | Garden | Seasonal-Perpetual | -35.13 | 0.16 | 0.16 |
|  |  | Seasonal-Mutant | -89.23 | 0.01 | <0.001 |
|  |  | Perpetual-Mutant | -54.10 | 0.07 | 0.07 |
| Date of Onset of Flowering | Garden | Seasonal-Perpetual | 50.99 | 2.52 | <0.001 |
|  |  | Seasonal-Mutant | 66.19 | 3.32 | <0.001 |
|  |  | Perpetual-Mutant | 15.20 | 3.17 | <0.001 |
| Date of End of Flowering | Garden | Seasonal-Perpetual | -18.01 | 4.80 | 0.002 |
|  |  | Seasonal-Mutant | -16.91 | 6.33 | 0.03 |
|  |  | Perpetual-Mutant | 1.11 | 6.04 | 1.00 |
| Date of Mean Flowering | Garden | Seasonal-Perpetual | 29.94 | 3.97 | <0.001 |
|  |  | Seasonal-Mutant | 36.95 | 5.23 | <0.001 |
|  |  | Perpetual-Mutant | 7.02 | 4.99 | 0.49 |
| Flowering Duration | Garden | Seasonal-Perpetual | -69.00 | 4.66 | <0.001 |
|  |  | Seasonal-Mutant | -83.10 | 6.14 | <0.001 |
|  |  | Perpetual-Mutant | -14.10 | 5.86 | 0.06 |
| DSDS50 | Garden | Seasonal-Perpetual | 32.94 | 2.99 | <0.001 |
|  |  | Seasonal-Mutant | 26.60 | 3.86 | <0.001 |
|  |  | Perpetual-Mutant | -6.30 | 3.92 | 0.35 |
| P50 | Garden | Seasonal-Perpetual | 3.73 | 0.33 | <0.001 |
|  |  | Seasonal-Mutant | 3.68 | 0.42 | <0.001 |
|  |  | Perpetual-Mutant | -0.05 | 0.43 | 1.00 |

**Table S5.** Summary data and pairwise differences for an ANOVA estimating 2015-17 survivorship in plants grown in the experimental garden by Accession Group.

| **ANOVA Model** |  |  |  |  |
| --- | --- | --- | --- | --- |
| **Factor** | ***F*** | **df** | ***p*** | **Partial eta^2^** |
| Model | 17.83 | 1 | 0.01 | 0.90 |
| Intercept | 79.24 | 1 | <0.001 | 0.95 |
| Accession Group | 17.83 | 1 | 0.01 | 0.90 |
|  |  |  |  |  |
| **Pairwise Comparisons** | **Estimate** | ***p*** |  |  |
| Seasonal-Perpetual | 81.18 | <0.001 |  |  |
| Seasonal-Mutant | 42.46 | <0.001 |  |  |
| Perpetual-Mutant | 38.72 | <0.001 |  |  |

**Table S6.** Summary data and pairwise differences for an ANCOVA estimating germination rate by years of natural aging and genotype (Pajares or *pep1-1*). Data is shown for naturally aged seeds.

| **ANCOVA Model** |  |  |  |  |
| --- | --- | --- | --- | --- |
| **Factor** | ***F*** | **df** | ***P*** | **Partial eta^2^** |
| Model | 66.13 | 5 | <0.001 | 0.97 |
| Intercept | 8103.75 | 1 | <0.001 | 0.99 |
| Year | 93.62 | 1 | <0.001 | 0.89 |
| Genotype | 99.52 | 2 | <0.001 | 0.94 |
| Year*Genotype | 21.94 | 2 | <0.001 | 0.79 |
|  |  |  |  |  |
| **Contrasts** |  |  |  |  |
| **Duration of Natural Aging** | **Mean Difference (Paj-*pep1-1*)** | |  |  |
| 48 Months | 33.28 | |  |  |
| 24 Months | 10.28 | |  |  |
| 12 Months | 8.01 | |  |  |

**Table S7.** Summary data for an ANOVA estimating germination rate by germination temperature and genotype (Paj or *pep1-1*). Data shown is for seed samples from long-term storage.

| **ANOVA Model** |  |  |  |  |
| --- | --- | --- | --- | --- |
| Factor | *F* | df | *p* | Partial eta^2^ |
| Model | 20.40 | 3 | 0.007 | 0.94 |
| Intercept | 1235.89 | 1 | <0.001 | 0.99 |
| Temperature | 2.67 | 1 | 0.18 | 0.40 |
| Genotype | 58.04 | 1 | 0.002 | 0.94 |
| Temperature*Genotype | 0.48 | 1 | 0.53 | 0.11 |
